# Transcriptional and Post-Translational Roles of Calcineurin in Cationic Stress and Glycerol Biosynthesis in *Cryptococcus neoformans*

**DOI:** 10.3390/jof10080531

**Published:** 2024-07-30

**Authors:** Ronaldo Silva Santos, Gabriel Martins-Silva, Adrián Adolfo Álvarez Padilla, Mateus Possari, Sérgio Donnantuoni Degello, Otávio J. Bernardes Brustolini, Ana Tereza Ribeiro Vasconcelos, Marcelo Afonso Vallim, Renata C. Pascon

**Affiliations:** 1 Universidade Federal de São Paulo, Campus Diadema, Rua São Nicolau, 210, Diadema 09913-030, SP, Brazil; ronaldosantos@icb.usp.br (R.S.S.); gabriel.martins21@unifesp.br (G.M.-S.); a.alvarezp27@gmail.com (A.A.Á.P.); matpossari.oliveira@usp.br (M.P.); sergio.degello12@unifesp.br (S.D.D.);; 2 Laboratório Nacional de Computação Científica—LNCC, Labinfo-Laboratório de Bioinformática, Petrópolis 25651-075, RJ, Brazil

**Keywords:** *Cryptococcus neoformans*, calcineurin, glycerol phosphate phosphatase, osmotic stress, cationic stress, *GPP*2

## Abstract

Stress management is an adaptive advantage for survival in adverse environments. Pathogens face this challenge during host colonization, requiring an appropriate stress response to establish infection. The fungal pathogen *Cryptococcus neoformans* undergoes thermal, oxidative, and osmotic stresses in the environment and animal host. Signaling systems controlled by Ras1, Hog1, and calcineurin respond to high temperatures and osmotic stress. Cationic stress caused by Na^+^, K^+^, and Li^+^ can be overcome with glycerol, the preferred osmolyte. Deleting the glycerol phosphate phosphatase gene (*GPP*2) prevents cells from accumulating glycerol due to a block in the last step of its biosynthetic pathway. Gpp2 accumulates in a phosphorylated form in a *cna*1Δ strain, and a physical interaction between Gpp2 and Cna1 was found; moreover, the *gpp*2Δ strain undergoes slow growth and has attenuated virulence in animal models of infection. We provide biochemical evidence that growth in 1 M NaCl increases glycerol content in the wild type, whereas *gpp*2Δ, *cna*1Δ, and *cnb*1Δ mutants fail to accumulate it. The deletion of *cnb*1Δ or *cna*1Δ renders yeast cells sensitive to cationic stress, and the Gfp-Gpp2 protein assumes an abnormal localization. We suggest a mechanism in which calcineurin controls Gpp2 at the post-translational level, affecting its localization and activity, leading to glycerol biosynthesis. Also, we showed the transcriptional profile of glycerol-deficient mutants and established the cationic stress response mediated by calcineurin; among the biological processes differentially expressed are carbon utilization, translation, transmembrane transport, glutathione metabolism, oxidative stress response, and transcription regulation. To our knowledge, this is the first time that this transcriptional profile has been described. These results have implications for pathogen stress adaptability.

## 1. Introduction

*Cryptococcus neoformans* has significant clinical importance. This encapsulated yeast is responsible for causing cryptococcosis, a potentially life-threatening fungal infection, particularly in AIDS patients, organ transplant recipients, or those undergoing immunosuppressive therapies [1].

As an opportunistic pathogen, *C. neoformans* exhibits a remarkable ability to respond to stress, such as high temperatures, high osmolarity, oxidative environments, nutrient abundance, and radiation, which are features intricately associated with its survival in nature but also associated with virulence in the host. The stress response depends on sophisticated cell signaling mechanisms that enable it to sense and respond to changes [2,3,4,5].

Calcineurin is pivotal in orchestrating fungal signaling, acting as a central hub that modulates adaptation to various environmental challenges [6]. As a calcium–calmodulin-activated protein phosphatase, calcineurin regulates critical cellular processes in fungi, such as ion homeostasis, cell wall integrity, growth, virulence, drug resistance, and response to stresses [7]. Upon exposure to stress or antifungal agents, calcineurin, a heterodimer composed of catalytic (Cna1) and regulatory (Cnb1) subunits, becomes activated by calcium–calmodulin. This complex binds to the catalytic subunit, exposing the active site of the autoinhibitory domain at the C-terminus; the Cnb1 subunit is also a Ca^+^-binding protein and considered a calcium sensor [8]. The active calcineurin complex leads to the dephosphorylation of target proteins and subsequent alterations in gene expression patterns [9]. This activation aids fungi in maintaining cellular integrity and viability under adverse conditions [8]. The most well-characterized role of the calcineurin complex in *C. neoformans* is in high-temperature growth at 37 °C [10,11]. It has been shown that cell exposure to inhibitors of the calcineurin complex, such as cyclosporine A, or the deletion of the regulatory and catalytic subunits leads to high-temperature growth failure and virulence attenuation in animal models of infection [3,12,13]. The transcription factor Crz1/Sp1 has been identified as the main target of the complex during stress [14] in *C. neoformans* and other fungi [6]. Calcineurin dephosphorylates Crz1 in the cytoplasm, which enters the nucleus to transcribe its target genes, leading to several cellular responses [15].

Crz1 has been found in stress granules (P-bodies), and Crz1 domains without previously attributed functions are linked to different localizations and functions [16]. However, the calcineurin complex has Crz1-independent roles: for example, Park et al. (2016) identified many calcineurin targets using a phosphoproteomic assay; besides Crz1, proteins involved in P-bodies/stress granules (PBs/SGs) were found as calcineurin substrates. Also, Cna1-Gfp co-localizes to PBs/SGs upon thermal stress, suggesting it may be involved in mRNA decay [17,18]. These observations are evidence that calcineurin is involved in a myriad of molecular processes to overcome stress. For example, Cna1 and Cnb1 subunits were found in a protein complex with the Cys3 transcription factor, a major regulator of sulfur uptake and amino acid biosynthesis that localizes mainly to the nucleus [19]. Along the same lines, Cna1 physically interacts with Met3 (ATP sulfurylase), a cytoplasmic protein involved in the first committed step of the sulfur uptake pathway, suggesting that the calcineurin complex contributes to nutritional stress regulation at multiple steps of the pathway [20].

Earlier, Cruz et al. (2000) observed cationic stress sensitivity (Na^+^ and Li^+^) in *cna*1Δ mutants of *C. neoformans*; conversely, *C. deneoformans* was not considered sensitive [10,12]. Cna1 and Cnb1 interact physically with Gpp2, the glycerol phosphate phosphatase responsible for glycerol biosynthesis, which is required to counteract cationic stress and promote cell wall integrity in *C. neoformans*; also, the *GPP*2 gene is required to activate virulence traits, according to previous observations [19,21,22]. In a phosphoproteome analysis, Gpp2 was found to be a calcineurin target. This protein accumulates in its phosphorylated form in a *cna*1Δ background [17]. This link between calcineurin and Gpp2 suggests a signaling mechanism for glycerol biosynthesis activation as a key physiological process in response to cationic stress. Our main hypothesis in this work is that calcineurin ultimately controls glycerol biosynthesis by modifying the Gpp2 protein at the post-translational level to promote cationic homeostasis. In this study, we show that calcineurin is required for proper Gpp2 localization at the subcellular level and glycerol biosynthesis. Also, by evaluating the global transcriptional profiles of *cna*1Δ, *cnb*1Δ, and *gpp*2Δ mutants, we reveal that the lack of calcineurin signaling and glycerol biosynthesis causes a large transcriptional rearrangement, promoting several cellular and physiological responses, including carbohydrate metabolism, cell wall and membrane remodeling, and glutathione and oxidative stress responses. Interestingly, cationic stress activates a set of 11 zinc-cluster-like transcriptional factors that are fungal-specific, several of which are dependent upon calcineurin signaling for transcriptional induction.

Given the critical role of glycerol metabolism in stress adaptation and virulence, understanding the interplay between calcineurin and Gpp2 not only sheds light on the pathogen’s strategies for thriving in various niches but also presents potential opportunities for devising novel therapeutic approaches against cryptococcosis.

## 2. Materials and Methods

### 2.1. Plasmids, Strains, and Primers

The plasmids, strains, and primers used in this work are listed in Supplementary Tables S1–S3, respectively.

### 2.2. Growth Conditions

Routine growth was carried out on YEPD (1% yeast extract, 2% bacto-peptone, 2% glucose). Synthetic dextrose (SD) was prepared with yeast nitrogen base, YNB (0.67 g/L yeast nitrogen base with or without amino acids and ammonium sulfate, depending on experimental design, 2% glucose). Growth was carried out at 30 °C unless specified otherwise. Growth in the liquid medium occurred at 150 rpm on a rotary shaker. Glutathione was supplemented at a 10 mM concentration. Multi-stress sensitivity was evaluated with YEPD medium supplemented with 0.07, 0,08, and 0.09 M LiCl or 0.5 and 1 M NaCl or KCl.

### 2.3. Strain Construction

This work used the TRACE (Transient CRISPR-Cas9 Coupled with Electroporation) method to create a *cnb*1Δ::*Neo*^R^ mutation in the wild-type strain H99, as previously published [23,24]. Briefly, pYF24 was used as a template to amplify the Cas9 endonuclease gene under the control of the *GPD*1 promoter by PCR. A 9Kb band was purified by QiaQuick (Qiagen, Hilden, Germany). EuPaGDT (Eukaryotic Pathogen CRISPR guide RNA/DNA Design Tool) embedded in the FungiDB site was used to design the gRNA target site (http://grna.ctegd.uga.edu/ accessed on 25 January 2021). The target site built was 20 nucleotides long, excluding the NGG sequence, to avoid self-cleavage of the gRNA. The nucleotide sequence selected as the target site was fused at its 3′ end to the sequences of the guide RNA scaffold to create the forward primer. A reverse primer for gRNA was also created, and the primer pair was used to amplify part of the guide RNA using the pDD162 (AddGene, Watertown, MA, USA) plasmid as the template [25]. The complementary sequence of the target site was incorporated into the reverse primer containing a sequence at its 3′ end complementary to sequences of the U6 promoter, and the forward primer to amplify the U6 promoter was also designed. The primer pair was used to PCR amplify the U6 promoter using *C. neoformans* strain JEC21 genomic DNA. A second PCR reaction, using the two PCR-amplified fragments as the template, was carried out to join the promoter and the gRNA sequence together using nested primers. This reaction generated a DNA fragment containing a 20-nucleotide target site fused to the guide DNA scaffold driven by the U6 promoter. This 300 bp fusion product was purified with QiaQuick. The *cnb*1Δ::*Neo*^R^ deletion construct containing 500 bp of the promoter and terminator regions of the *CNB*1 gene flanking the *Neo*^R^ resistance gene driven by the actin promoter was made by overlapping PCR, as previously described [26]. All three DNA fragments encoding the gRNA, Cas9, and deletion construct were purified, eluted in water, and introduced into the wild-type strain H99 by electroporation according to the protocol previously published [24]. Transformants resistant to G418 were subjected to 5 cycles of subculture in YEPD without antibiotics to eliminate transient transformants. Finally, the transformants were inoculated in YEPD supplemented with G418. They were also subjected to diagnostic PCR to identify homologous recombination at the *CNB*1 locus. Among forty transformants, six were positive in the diagnostic PCR (15% of homologous integration) and were designated CNU160 to CNU165. All of them were temperature-sensitive at 37 °C, a marked phenotype of the calcineurin mutants. CNU160 was chosen for further analysis.

Strains containing the *GFP-GPP*2 allele were constructed by PCR amplification of the *GPP*2 gene from the Start to Stop codon plus 300 bp of the terminator region. gDNA of the H99 wild-type strain was used as a template with PRCP472 and PRCP473 primers (Supplementary Information, Table S3). The band was excised from agarose gel, and the DNA was cloned into the pCN19 plasmid and digested with *Bam*HI and *Spe*I restriction enzymes. Gibsom assembly (NEB) was used to reconstitute the circular plasmid containing the *GPP*2 gene. The vector contains the *GFP* sequence driven by the Histone H3 promoter and the nourseothricin resistance gene as a selectable marker. The plasmid was sequenced to confirm translational fusion between *GFP* and *GPP*2 sequences. After confirmation, the plasmid (pRCP115) was introduced into the wild-type strain H99 (CNU151) and into the mutant strains CNU160 (*cnb*1Δ) and CNU166 (*cna*1Δ), generating the strains CNU189 and CNU193, respectively.

### 2.4. Western Blot

The protein extract was obtained and separated by SDS-PAGE, as described before [19]. The gels were equilibrated in transfer buffer (48 mM Tris, 39 mM glycine, 20% methanol), and proteins were transferred to nitrocellulose membranes on the Trans-Blot^®^ SD Semi-Dry Electrophoretic Transfer Cell (BioRad, Hercules, CA, USA) at 15 V for 1 h. The membrane was blocked with 5% non-fat dry milk in TBS (10 mM Tris, 150 mM NaCl, pH 7.4) for 1 h at room temperature. The primary antibody (mouse anti-GFP ThermoFisher (Waltham, MA, USA), 1:7000 dilution) was incubated overnight at 4 °C in 1% BSA. After three washes, 5 min each, in TBST (TBS with 0.1% Tween 20), the secondary antibody (goat anti-mouse-HRP, Cell Signaling Technology 1:2000 dilution) was incubated in TBST with 5% non-fat dry milk for 1 h at room temperature, followed by three 5 min washes, each in TBST. SuperSignal West Pico PLUS Substrate (ThermoFisher) was used with the ImageQuant LAS 4000 system (GE) to detect chemiluminescent bands. Loading control was performed with the rabbit anti-Histone H3 antibody (1:2000) and anti-rabbit HRP-linked secondary antibody (1:2000). Protein extraction and Western blotting were performed in triplicate.

### 2.5. Fluorescence Microscopy

Strains containing the *GFP-GPP*2 allele were induced in several stress conditions, depending on the experimental design. Cells were grown overnight in 5 mL of YEPD at 30 °C with 150 rpm rotation, washed with PBS three times, then diluted to OD_600_ = 0.6 (5 mL), and incubated under salt stress for two hours or with a calcineurin inhibitor for 0, 2, or 4 h. A 1 mL aliquot was removed for microscopy analysis. The cells were fixed in 4% formaldehyde (Sigma, St. Louis, MO, USA) (*v*/*v*) diluted in 100 mM potassium phosphate and 0.5 mM MgCl_2_ for 10 min at 30 °C and washed twice with 1× PBS. Glass slides were prepared with 4 µL of ProLong with NucBlue antifade (Thermo Scientific, Waltham, MA, USA) and 6 µL of the processed sample. Cells were viewed by direct fluorescence microscopy using an Olympus BX51M microscope (Tokyo, Japan), and an analysis was performed using Olympus CellSens 1.15 and PhotoShop CS6 13.0. All microscopy experiments were performed in triplicate.

### 2.6. RNA Extraction

Liquid cultures of H99, CNU166 (*cna*1Δ::*Hph*^R^), CNU160 (*cnb*1Δ::*Neo*^R^), CNU125 (*gpp*2Δ::*Nat*^R^, Gfp-Cys3), and CNU080 (pCN50/*Neo*^R^—Gfp-Cys3) were grown overnight in 50 mL of YEPD at 30 °C, 150 rpm. Cells were collected and washed 2 times in 1× PBS by centrifugation at 4000 rpm for 10 min at room temperature. Cells were inoculated at OD_600_ = 0.5 in 15 mL of YEPD with and without 5 mM H_2_O_2_ or 1 M NaCl for 30 min with 150 rpm rotation at 30 °C. After this period, the cells were collected and washed in DEPC-treated water, and the pellets were lyophilized overnight. According to the manufacturer’s instructions, the RNAs were extracted with a Yeast RNeasy Mini Kit (Qiagen). The RNAs were quantified in Nanodrop (Thermo Scientific) and used in RNA-seq or qPCR.

### 2.7. RNA-Seq and Bioinformatics

RNA-seq libraries were prepared from 500 ng of total RNA using the TruSeq Stranded mRNA Sample Preparation Kit (Illumina, San Diego, CA, USA), following the manufacturer’s protocol without any modifications. Library QC and quantification were performed using the High Sensitivity D1000 ScreenTape Assay on the 4200 TapeStation system (Agilent, Santa Clara, CA, USA). Sequencing was performed in a NextSeq 500 sequencing system (Illumina) set to obtain 2 × 75 bp reads.

The sequencing files in fastq format were processed with BBDuk version 39.01 (https://doi.org/10.1371/journal.pone.0185056 accessed on 18 August 2023) to filter out adapters and low-quality reads. Quality reports were generated using FASTQC version 0.12.1 (https://www.bioinformatics.babraham.ac.uk/projects/fastqc accessed on 18 August 2023). Read mapping was conducted using STAR software version 2.7.10b against the *C. neoformans* var. grubii H99 genome. To generate the count table, read counts were quantified with the feature Counts program version 2.0.6 (https://doi.org/10.1093/bioinformatics/btt656 accessed on 18 August 2023). Differentially expressed genes (DEGs) were identified using DESeq2 (https://doi.org/10.1186/s13059-014-0550-8 accessed on 18 August 2023), applying an adjusted *p*-value cutoff of 0.05 and a log2 fold change threshold of 1. Annotation data were retrieved from the FungiDB database (https://doi.org/10.3390/jof4010039 accessed on 18 August 2023) and EnsemblFungi (https://fungi.ensembl.org/Cryptococcus_neoformans_var_grubii_h99_gca_000149245 accessed on 18 August 2023). The R 4.1 and Bioconductor 3.16 package GOstats (https://doi.org/10.1093/bioinformatics/btl567 accessed on 18 August 2023) was used to perform the Gene Ontology (GO) enrichment analysis, and pathview (https://doi.org/10.1093/bioinformatics/btt285 accessed on 18 August 2023) was used for the KEGG analysis.

### 2.8. Quantitative PCR

The Platus transcriber RNAse H—cDNA First Strand kit (Sinapse Inc., São Paulo, SP, Brazil) was used for cDNA synthesis from 5 µg of total RNA together with Oligo(dt) and Random hexamer primers. Diluted cDNA templates (1:10) were amplified with 100 ƞM of the target primers, 300 ƞM of the endogenous control *GPDH*1 (Glyceraldehyde-3phosphate dehydrogenase), and the 1× Syber green master mix (Evagreen^®^). The quantification of transcript levels was performed in the StepOnePlus™ Real-time PCR System thermocycler (Waltham, MA, USA) using the 2^ΔΔCT^ method and normalized against *GPDH*1 as previously described [27]. An analysis of variance was performed using the Tukey multiple-comparison test using Prism Graphpad 8.0 software, and *p* values lower than 0.05 were considered statistically significant.

### 2.9. Glycerol Quantification

Intracellular glycerol determination was performed as previously reported [28,29] with modifications. Cells were grown overnight in YPD medium at 30 °C. The following day, cultures were diluted in fresh YPD medium to obtain an OD600 nm of 0.2 and grown in YPD medium with or without supplementation with 1 M NaCl at 30 °C to the mid-log phase (OD600 nm of 0.7, approximately). Then, cells were collected by centrifugation (1 min at 6000× *g*), resuspended in 1 mL of boiling water, and incubated at 100 °C for 10 min. The samples were cooled on ice for 10 min and then centrifuged at 15,000× *g*. Supernatants were used to measure glycerol and were stored at −20 °C. The glycerol concentration was determined using a commercial kit (Glicerol Assay kit) following the manufacturer’s instructions (MAK117, Sigma Aldrich, St. Louis, MO, USA).

## 3. Results

### 3.1. gpp2 Δ, cna1 Δ, and cnb1 Δ Mutants Are Sensitive to Cationic Stress and Fail to Accumulate Glycerol

**Figure 1:**
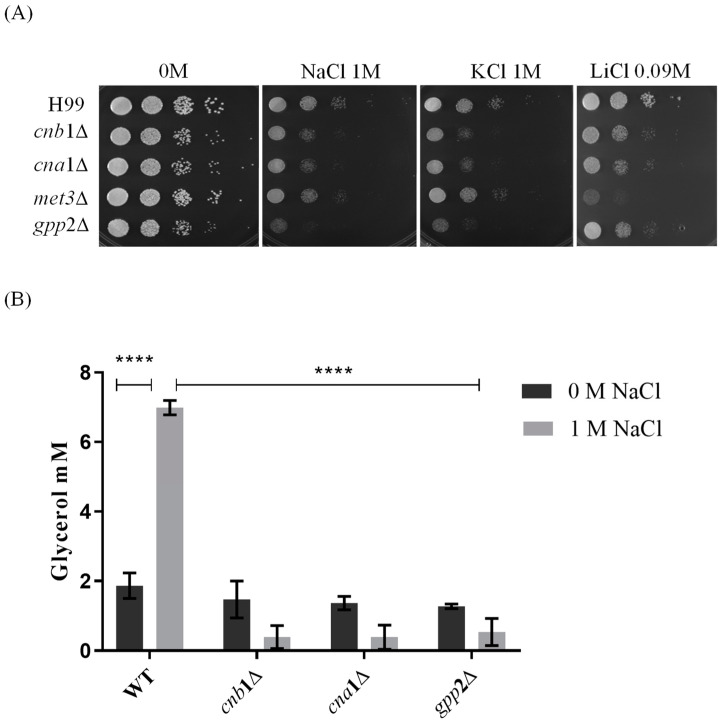
Sensitivity to cationic stress in *cnb*1Δ, *cna*1Δ, *met*3Δ, and *gpp*2Δ mutant strains compared to the wild type (H99). (**A**) The growth phenotype of wild-type and mutant strains cultures in YEPD medium containing NaCl, KCl (1 M each), and LiCl (0.09 M). The plates were incubated at 30 °C for 48 h. (**B**) The quantification of intracellular glycerol in *cna*1∆, *cnb*1∆, and *gpp*2∆ mutant and wild-type H99 strains. The growth of wild-type and mutant strains occurred in YPD medium with (gray bars) and without (black bars) 1 M NaCl supplementation at 30 °C overnight at 150 rpm. A two-way ANOVA multiple-comparison test was used to find relevant statistical differences (*p* value < 0.0001 ****).

Reports from our group have shown that cationic homeostasis depends upon Gpp2 phosphatase and that glycerol supplementation of the medium remediates cationic stress sensitivity in the *gpp*2Δ strain [21]. Also, our previous work and others have shown that Gpp2 is a target of the calcineurin complex, and it physically interacts with Cna1ΔC and Cnb1 [17,19]. Based on these observations, we hypothesized that the calcineurin complex controls cationic stress, at least partially, by activating the Gpp2 protein and glycerol biosynthesis. In order to challenge this hypothesis, first, we tested whether *cna*1Δ and *cnb*1Δ strains would show growth deficiency under cationic stress (NaCl, KCl, and LiCl). Also, we added *gpp*2Δ and *met*3Δ (ATP sulfurylase) mutants to this experiment [20,21]. The first was a positive control since we know it is hypersensitive to NaCl and KCl, and the latter was added to the analysis because ATP sulfurylase physically interacts with Cna1. Therefore, the mutants’ growth may also be affected by cationic stress. Figure 1A shows that all strains tested are sensitive to 1 M NaCl and KCl and 90 mM LiCl compared to the wild type to different degrees. Notably, the *gpp*2Δ and *met*3Δ strains are hypersensitive to NaCl/KCl and LiCl, respectively, whereas both calcineurin mutants are sensitive to all salts used. This result suggests that calcineurin is involved in cationic homeostasis, probably by, directly or indirectly, causing post-translational modifications in the Gpp2 protein.

Since Gpp2 is responsible for the last step in glycerol biosynthesis and, theoretically, its deletion would lead to low intracellular glycerol, we carried out glycerol quantification in the wild type, *cna*1Δ, *cnb*1Δ, and *gpp*2Δ in the presence or absence of NaCl. Figure 1B shows that in the wild-type strain (H99), the glycerol content increases significantly in the presence of NaCl; however, all three mutants fail to accumulate glycerol in the presence of NaCl.

Taken all together, these results indicate that glycerol is an important osmolyte during cationic stress, and its biosynthesis and accumulation are dependent on Gpp2, a glycerol phosphate phosphatase, which may be activated by the calcineurin signaling complex.

### 3.2. Cna1 and Cnb1 Subunits Are Required for Proper Gpp2 Protein Integrity and Localization to Cytoplasmic Puncta

**Figure 2:**
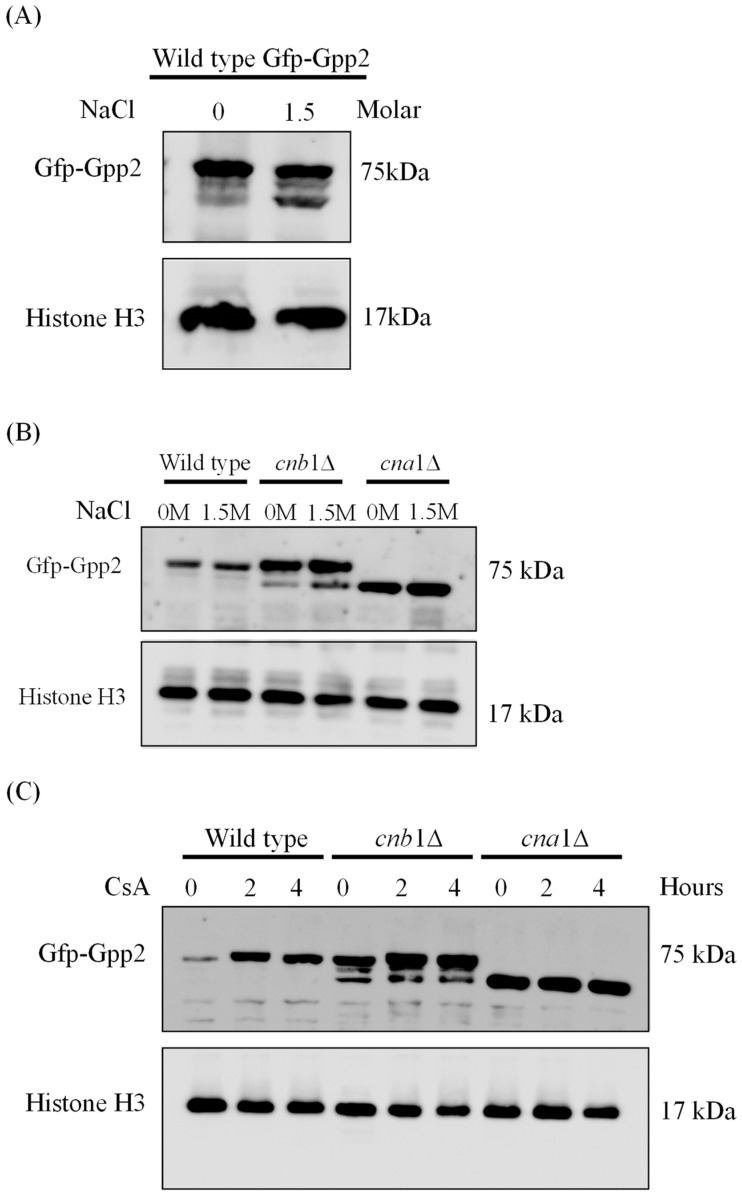
The Western blotting expression pattern of the Gfp-Gpp2 protein. (**A**) A Western blot of total proteins extracted from the wild-type strain (CNU151) expressing the *GFP*-*GPP*2 allele cultivated for 2 h in SD + N (30 °C) in the presence or absence of 1.5 M NaCl. (**B**) A Western blot of total proteins extracted from the wild-type strain (CNU151) and calcineurin mutant strains (CNU189, *cnb*1Δ, and CNU193 *cna*1Δ ) expressing the *GFP*-*GPP*2 allele cultivated for 2 h in SD + N (30 °C) in the presence or absence of 1.5 M NaCl. (**C**) A Western blot of total proteins extracted from the wild-type strain (CNU151) and calcineurin mutant strains (CNU189, *cnb*1Δ, and CNU193 *cna*1Δ) treated with 100 µg/mL of cyclosporin A (CsA) for 0, 2, or 4 h in SD + N (30 °C). The mouse anti-GFP primary antibody was used at a 1:7000 dilution, and the anti-mouse horseradish peroxidase-linked secondary antibody was applied at a 1:2000 dilution. Loading control was performed by the detection of the Histone H3 protein with the rabbit anti-His3 primary antibody (1:2000) and anti-rabbit horseradish peroxidase-linked secondary antibody (1:2000).

Since calcineurin is a serine/threonine phosphatase, we checked whether a Gfp-Gpp2 fusion protein would be modified by deleting the genes encoding the regulatory (*CNB*1) and catalytic (*CNA*1) calcineurin subunits. First, Western blotting was carried out in the absence or presence of NaCl in the wild-type (CNU151) strain containing the *GFP*-*GPP*2 allele. As shown in Figure 2A, a 75 KDa band was observed, as expected from the theoretical molecular weight calculated for the fusion protein. In the presence of 1.5 M NaCl, besides the main band at 75 KDa, a lower-molecular-weight band was detected in response to NaCl added to the medium (Figure 2A). The same behavior can be observed for the Gfp-Gpp2 fusion protein in the *cnb*1Δ strain (Figure 2B). However, in the *cna*1Δ strain, the 75 KDa band was abolished, and only the lower-molecular-weight band was detected (Figure 2B). This result suggests that the Cna1 catalytic subunit is required to maintain the full-length Gpp2. Its absence seems to lead to Gfp-Gpp2 being processed to a lower-molecular-weight band, estimated at 60 KDa. Also, the wild-type and mutant strains (cna1Δ and cnb1Δ) containing the *GFP*-*GPP*2 allele were exposed to 100 µg/mL of cyclosporine A, which was previously described as a calcineurin inhibitor [30], and Western blotting was performed. Figure 2C shows that the inactivation of calcineurin in the wild-type strain leads to a progressive increase in Gfp-Gpp2 accumulation after 2 and 4 h post-induction. The same can be seen for the cnb1Δ strain, where the main 75 KDa band accumulates in larger amounts after 2 and 4 h; in addition, the cnb1Δ strain has the 60 KDa band in higher amounts as well. CsA had no observable effect on the Gfp-Gpp2 band in the cna1Δ background strain. This result suggests that Gpp2 is a target of calcineurin-mediated post-translational modifications.

**Figure 3:**
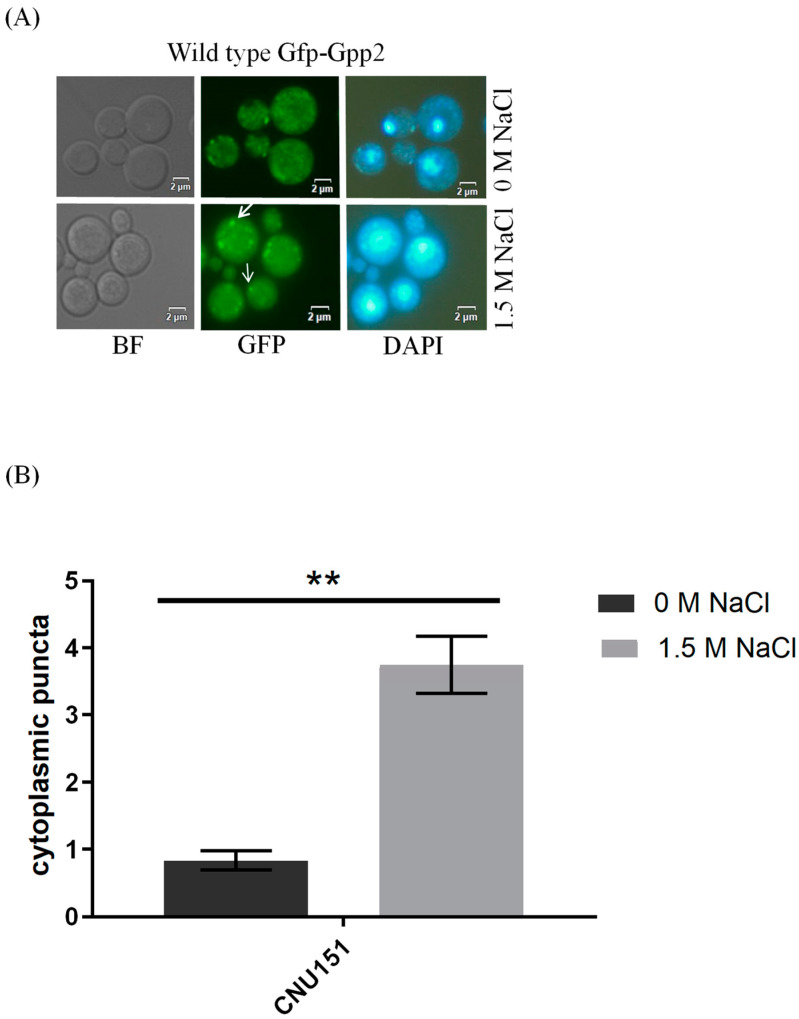

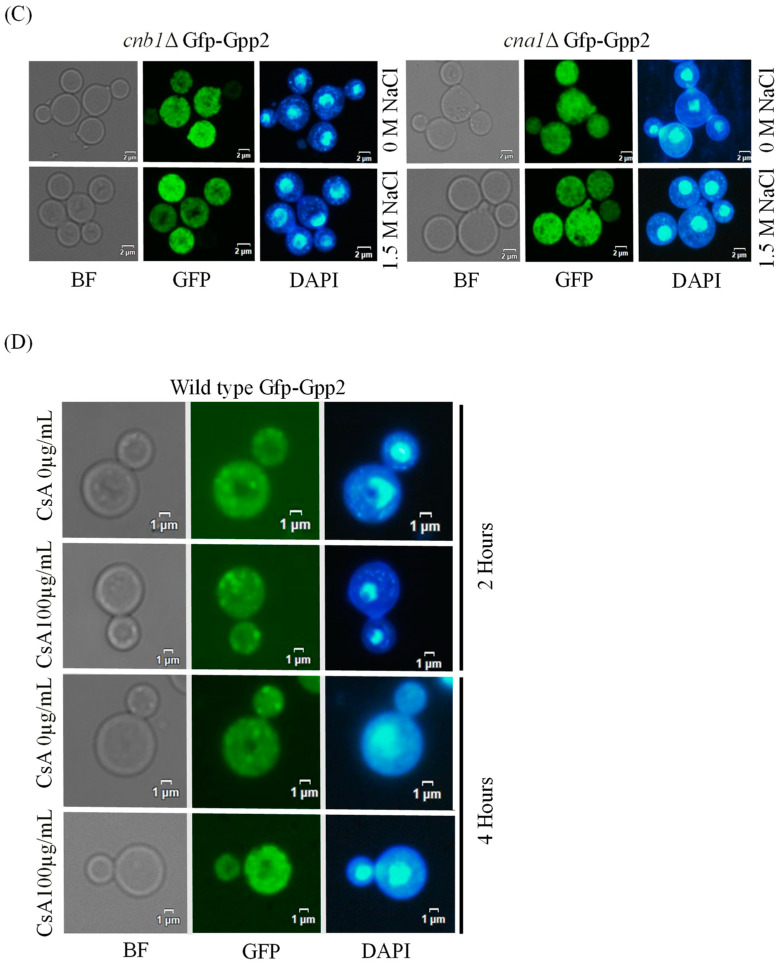
Gfp-Gpp2 protein localization observed by fluorescence microscopy: (**A**) subcellular localization in the wild-type strain (CNU151) with and without cationic stress (1.5 M NaCl); (**B**) cytoplasmatic puncta per cell (n > 100) in the wild-type strain (CNU151) without (black bar) and with cationic stress (gray bar) (1.5 M NaCl). *p* value < 0.05 (**). (**C**) Gfp-Gpp2 localization in calcineurin mutants (CNU189, *cnb*1Δ, and CNU193 *cna*1Δ) with and without cationic stress (1.5 M NaCl); (**D**) wild-type strain (CNU151) treated for 0, 2, or 4 h with cyclosporin A (SD + N + 100 µg/mL CsA, 30 °C). Bright-field = BF; GFP fluorescence = GFP; and nuclear staining = DAPI.

Fluorescence microscopy was performed to detect the subcellular localization of Gfp-Gpp2 in the same conditions as the Western blots. In the wild type, Gfp-Gpp2 is distributed homogeneously in the cytoplasm. However, after 2 h, in the presence of NaCl, the Gfp-Gpp2 protein accumulated in distinct punctual dots in the cytoplasm (Figure 3A). These cytoplasm puncta increased from an average of one to more than four puncta per yeast cell in the presence of NaCl (Figure 3B). Fluorescence microscopy of the mutant strains *cna*1Δ and *cnb*1Δ carrying the *GFP*-*GPP*2 allele showed no particular organization as cytoplasmic puncta, either in the absence or in the presence of NaCl (Figure 3C). The effect of cyclosporine A was analyzed, and after 4 h in the presence of NaCl and 100 µg/mL of CsA, the Gfp-Gpp2 puncta localization was lost (Figure 3D). The data suggest that the Gfp-Gpp2 protein undergoes post-translational modifications and distinct localization during cationic stress; both processes depend on calcineurin.

### 3.3. Global Transcriptional Profiles of the gpp2Δ, cna1Δ, and cnb1Δ Strains under Cationic Stress

Our data support that calcineurin contributes to the cationic stress response by causing Gpp2 protein modifications and altering its spatial localization, culminating in glycerol biosynthesis. These experiments established a post-translational role for calcineurin in glycerol biosynthesis; however, there is no insight into the global transcriptional profile of the wild-type strain H99 in response to cationic stress induced with 1 M NaCl and how calcineurin would affect this stress response at the transcriptional level in *C. neoformans*. Therefore, a comparison among the *cna*1Δ, *cnb*1Δ, and *gpp*2Δ mutants in 1 M NaCl was conducted to broaden our understanding and possibly find a mechanism by which the cationic stress response happens at the transcriptional level. Also, since all three mutants are deficient in glycerol accumulation, we aimed to relate the global transcriptional response to a lack of glycerol.

As part of the transcriptome validation, we measured the gene expression levels of *GPP*2 (CNAG_01744 = −3.99 log2 FC), *CNA*1 (CNAG_04796 = −4.80 log2 FC), and *CNB*1 (CNAG_00888 = −0.54 log2 FC), which were found repressed in the *gpp*2Δ, *cna*1Δ, and *cnb*1Δ strains, respectively, as expected. Also, *GPP*2 (CNAG_01744) and *GPP*1 (CNAG_06698) were up-regulated under cationic stress (0.83 and 0.91 Log2 FC, respectively), as reported earlier [2].

**Figure 4:**
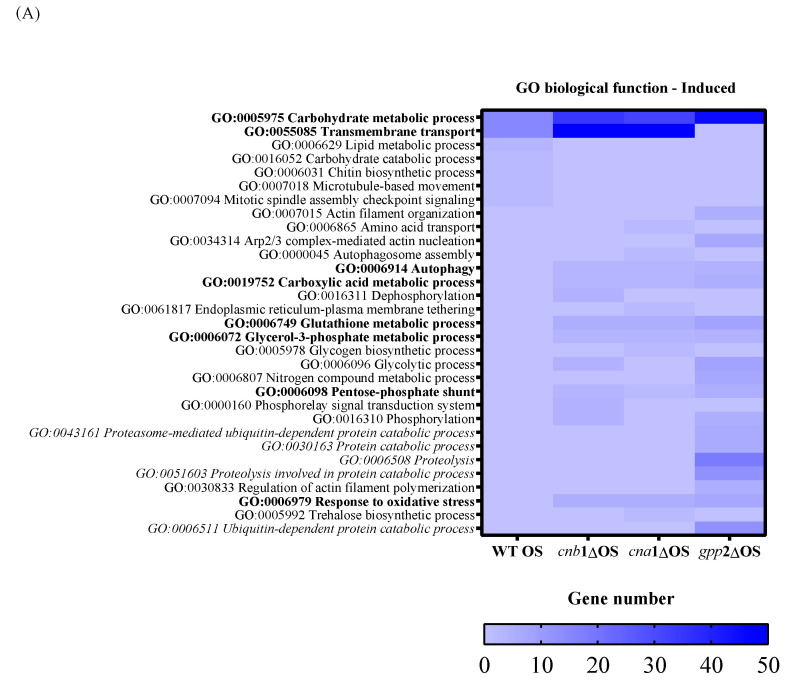

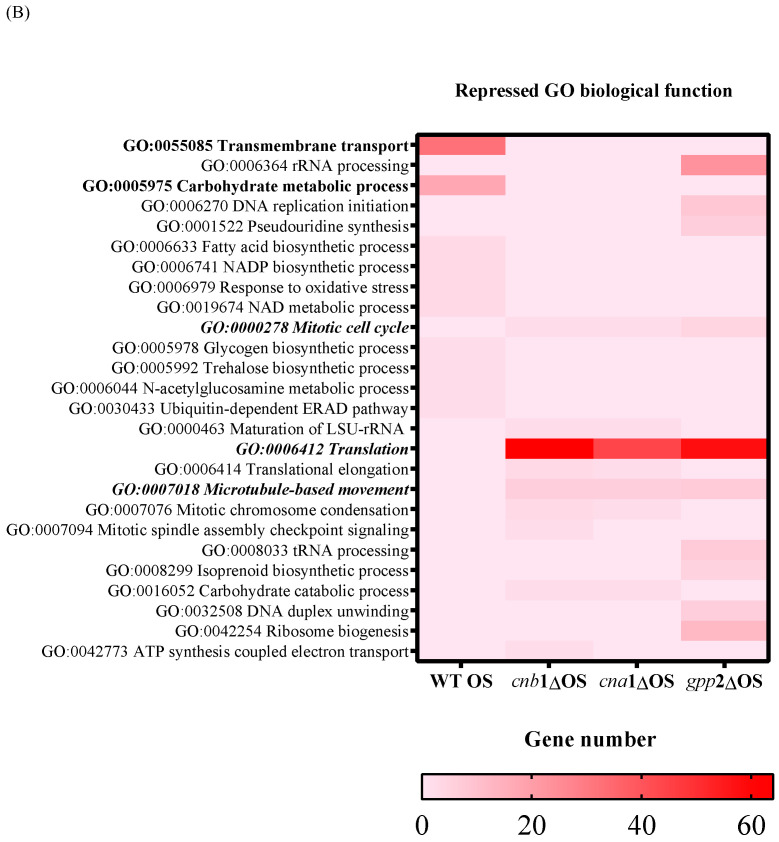
A heat map of GO according to biological function in the wild type and mutants (*cna*1Δ, *cnb*1Δ, *gpp*2Δ). In (**A**) are the induced categories (in blue) during cationic stress, and in (**B**) are the repressed categories (in pink); *p* value < 0.05. More intense colors represent a higher number of genes in each category.

To characterize the global gene expression profile during cationic stress, we first analyzed the wild-type (H99) response to NaCl and then compared it to the mutants. Regarding the number of DEGs in each comparison, the wild-type strain showed 433 up-regulated and 544 down-regulated DEGs in the presence of cationic stress (Table 1). As for the calcineurin mutants, the *cnb*1Δ and *cna*1Δ strains presented 815 and 760 up-regulated DEGs, respectively, and 376 and 315 down-regulated DEGs, respectively; the *gpp*2Δ strain showed the largest number of DEGs (1396 up-regulated and 1226 down-regulated), as presented in Table 1. Figure 4A,B show the heat maps of induced (in blue) and repressed (in red) DEGs by GO biological function. Regarding induced categories (heat map in blue, Figure 4A), the carbohydrate metabolic process (GO: 0005975) is the only induced biological function common to all four strains under cationic stress. Transmembrane transport (GO:0055085) is the second category with the highest number of induced DEGs (Figure 4A in blue) in the wild type and calcineurin mutants. Only the wild-type strain presented induced genes categorized under lipid metabolic process, carbohydrate catabolic process, chitin biosynthetic process, microtubule-based movement, and mitotic spindle assembly checkpoint signaling (GO:0006629, GO:0016052, GO:0006031, GO:0007018, and GO:0007094, respectively). Biological functions with the highest number of DEGs induced specifically in all three mutants are autophagy, carboxylic acid metabolic process, glutathione metabolic process, glycerol-3-phosphate metabolic process, pentose-phosphate shunt, and response to oxidative stress (GO:0006914, GO:0019752, GO:0006749, GO:0006072, GO:0006098, and GO:0006979, respectively, Figure 4A). Biological functions specifically induced in the *gpp*2Δ mutant are related to protein catabolism (highlighted in italics): ubiquitin-dependent protein catabolic process, proteosome-mediated ubiquitin-dependent protein catabolic process, protein catabolic process, proteolysis, and proteolysis involved in protein catabolic process (GO:0006511, GO:0043161, GO:0030163, GO:0006508, and GO:0051603, respectively Figure 4A).

In summary, three important elements arise from this analysis: (i) Carbon metabolism remodeling is important for counteracting cationic stress in all strains, probably by releasing energy and by changing cell wall composition; however, the carbon metabolism change does not seem to be controlled by calcineurin signaling, but indeed represents an important response to control damage caused by cationic stress. (ii) Transmembrane transport seems to be an important response to cationic stress for the wild type and calcineurin mutants, which may use metabolite transport to maintain homeostasis; however, *gpp*2Δ does not respond by enhancing membrane transport and instead seems to activate protein catabolism, proteolysis, and ubiquitin-dependent proteolysis as a way to handle misfolded proteins due to ionic imbalance. This difference in transcriptional response suggests that Gpp2 may have additional roles in cationic stress that are not related to glycerol biosynthesis since all three mutants have very similar glycerol content. (iii) The response to oxidative stress seems to play an important role in cationic stress for all three mutants, but not for the wild type.

Regarding the down-regulated genes, in the wild type under cationic stress, repressed biological functions categorized by GO are carbohydrate metabolism and transmembrane transport (Figure 4B, heat map in red). Genes categorized under translation, rRNA biogenesis, mitotic cell cycle, and microtubule movement were repressed in all three mutants, with an emphasis on genes related to ribosomal biogenesis and translation (ribosomal proteins mostly), suggesting that the absence of calcineurin signaling and low glycerol content led to growth arrest. Even though the transcriptional response seems more complex in the *gpp*2Δ mutant, the transcriptional profiles of the three mutants are similar (Figure 4B).

### 3.4. Glycerol-3-Phosphate Metabolic Flow Is Altered in Calcineurin and gpp2 Mutants

**Figure 5:**
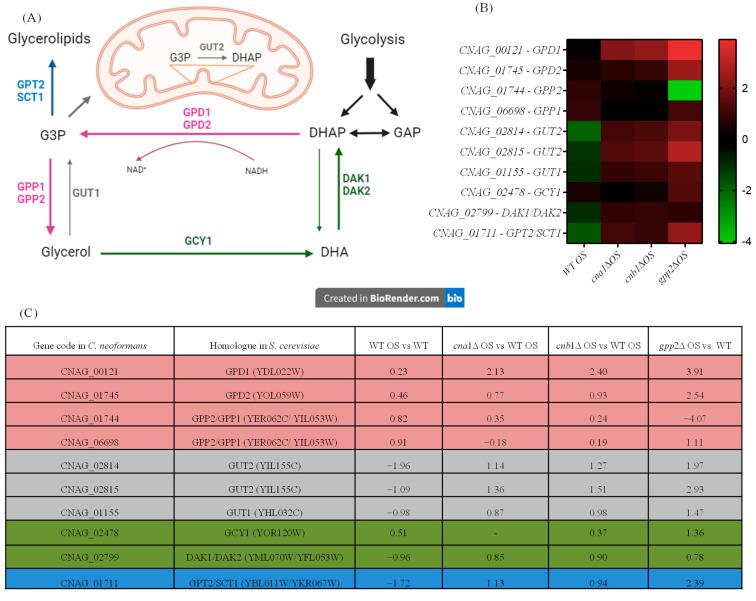
Gene expression of genes in the glycerol biosynthetic pathway (GO:0006072). (**A**) Genes, proteins, and metabolic intermediates involved in glycerol biosynthesis; (**B**) heat map of the DEGs involved in glycerol biosynthesis in the wild type and mutants; (**C**) glycerol biosynthesis gene codes in *C. neoformans* and *S. cerevisiae* and fold changes in expression in the wild type and mutants.

Since glycerol accumulation is affected by calcineurin and Gpp2 mutations (Figure 1B) and glycerol-3-phosphate metabolism (GO:0006072) is a biological process altered in the transcriptome during cationic stress (Figure 4A), the expression levels of differentially expressed genes (DEGs) in this pathway were evaluated (Figure 5A). As shown in Figure 5A,B, in the wild-type strain under cationic stress (WT OS), the metabolic flow is directed toward glycerol biosynthesis by a slight increase in gene expression of the GPD1/2 and GPP1/2 genes, whereas the genes (*GUT*1 and *GUT*2) involved in generating dyhydroxyacetone phosphate (DHAP) and glycerol-3-phosphate (G3P) are repressed (Figure 5B,C); likewise, the transformation of glycerol to DHA and DHAP by *GCY*1 and *DAK*1/2, respectively, is also repressed, since these genes are down-regulated (Figure 5B,C). Finally, *GPT*2/*SCT*1, which is the first committed step of G3P conversion to glycerolipids, is down-regulated. These observations suggest that glyceraldehyde phosphate (GAP) and glycolysis are not favored in this condition; along the same lines, the metabolic flow is not directed toward glycerolipid biosynthesis. In summary, during cationic stress, the transcriptional response of the wild-type strain is directed toward glycerol biosynthesis (pink arrows in Figure 5A).

The *cna1*Δ, *cnb1*Δ, and *gpp2*Δ strains have similar expression profiles, as shown in the heat map (Figure 5B). Glycerol biosynthesis is no longer favored in the calcineurin mutants since the GPP1/GPP2 genes are down-regulated in these strains (*cna1*Δ and *cnb1*Δ); however, GPD1, which is necessary to generate glycerol-3-phosphate (G3P), is up-regulated in all three mutants (Figure 5A, pink arrow), increasing G3P biosynthesis; the *GUT*1 and *GUT*2 genes are also highly induced in all three mutants, especially *GUT*2, suggesting that G3P is being used to generate DHAP. The conversion of glycerol to DHA and DHAP is also repressed since the GCY1 and DAK1/DAK2 genes are repressed in the calcineurin mutants, but they are induced in the gpp2Δ strain (Figure 5A, green arrows). The branch of the pathway that leads to glycerolipids from G3P is activated since the GPT2 gene is up-regulated in all three mutants.

In summary, during cationic stress, the wild type channels the key metabolites in this pathway (G3P and DHAP) to glycerol biosynthesis, and the mutants channel it to gluconeogenesis and glycerolipid biosynthesis. Also, this result suggests that calcineurin not only controls this pathway at the post-translational level by modifying Gpp2 protein localization and processing but also may act at the transcriptional level, modulating the glycerol biosynthesis regulatory circuit.

In summary, during cationic stress, the wild type channels the key metabolites in this pathway (G3P and DHAP) to glycerol biosynthesis, and the mutants channel it to gluconeogenesis and glycerolipid biosynthesis. Also, this result suggests that calcineurin not only controls this pathway at the post-translational level by modifying Gpp2 protein localization and processing but also may act at the transcriptional level, modulating the glycerol biosynthesis regulatory circuit.

### 3.5. Lack of Glycerol Modulates Carbon Metabolism, Translation, Transmembrane Transport, and Protein Degradation at the Transcriptional Level

**Figure 6:**
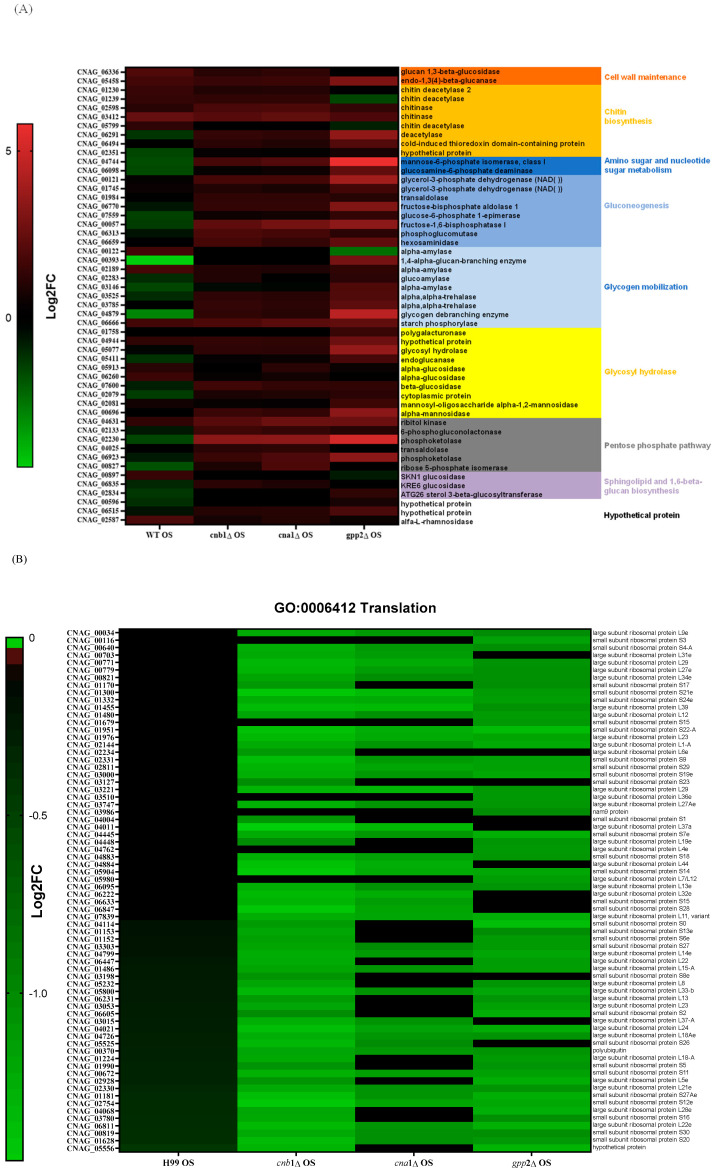

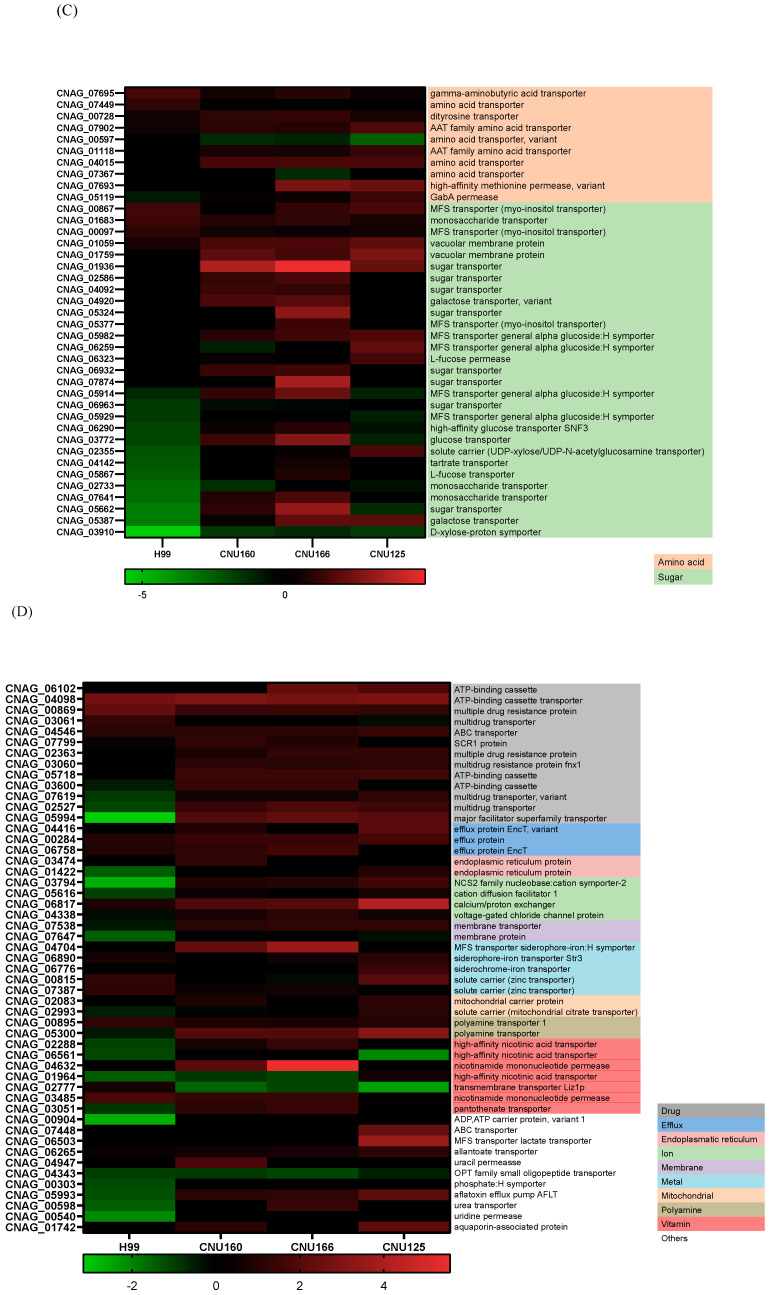
(**A**) A heat map of DEGs involved in the carbohydrate metabolic process (GO:0005975) in the wild type and mutants exposed to cationic stress; (**B**) a heat map of DEGs involved in translation (GO:0006412) in the wild type and mutants exposed to cationic stress; (**C**,**D**) a heat map of DEGs involved in transmembrane transport (GO:0055085) in the wild type and mutants exposed to cationic stress.

Since carbohydrate metabolism (GO: 0005975) is a GO category that seems important for the cationic stress response, we analyzed the DEGs in this category in mutant and wild-type strains in NaCl. The wild-type strain showed more repressed genes (19 genes in green, first column, Figure 6A) than the mutants; the *cna*1Δ and *cnb*1Δ strains had just 1 repressed DEG, while the *gpp*2Δ strain presented 4 repressed DEGs. Figure 6A shows that the DEGs in this category (GO:0005975) are involved in cell wall remodeling and chitin metabolism (red and orange letters), gluconeogenesis (medium blue), glycogen mobilization (light blue), glycosyl hydrolases (yellow), pentose phosphate pathway (gray), sphingolipid and sterol biosynthesis (lilac), and amino sugar and nucleotide sugar metabolism (dark blue). In general, wild-type and calcineurin-deficient strains were able to remodel their cell walls by enhancing the expression of glucan 1,3-beta-glucosidase and endo-1,3(4)-beta-glucanase (CNAG_06336 and CNAG_05458); the first is a major protein required for cell wall biosynthesis (*BGL*2, YGR282C in *S. cerevisiae*), involved in the incorporation of newly synthesized mannoprotein molecules into the cell wall in *S. cerevisiae* [31,32], and the second codes for a glycosyl hydrolase important for the spore wall, required for normal spore wall assembly (*CRR*1, YLR213C in *S. cerevisiae*), possibly for cross-linking the glucan and chitosan layers [33]. Most chitin deacetylases and chitinases were also induced or unaffected in all strains, except in *gpp*2Δ, where two of them were repressed (Figure 6A). All of the remaining DEGs were induced in the calcineurin-deficient and *gpp*2Δ strains and repressed or unaffected in the wild type, especially gluconeogenesis, glycogen mobilization, glycosyl hydrolases, pentose phosphate pathway, nucleotide sugar metabolism, and sphingolipid and sterol metabolism (Figure 6A). The induction of these genes suggests that the energetic mobilization of reserve carbohydrates is more pronounced in mutants than in the wild-type strain under cationic stress, which is in agreement with the metabolic flow channeling G3P and DHAP to gluconeogenesis and glycerolipid biosynthesis, as shown in Figure 5.

Translation (GO:006412) is a highly repressed category in the *cnb*1Δ, *cna*1Δ, and *gpp*2Δ strains compared to the wild type. Sixty-four DEGs grouped in this GO category encode ribosomal proteins, which are highly repressed in the mutants (Figure 6B). Seventy genes encoding mostly large and small subunit ribosomal proteins were unaffected in the wild type and highly repressed in the mutants, as shown. This result is in agreement with the low growth observed in the mutants compared to the wild type in 1 M NaCl (Figure 1A), suggesting that the inability to achieve osmobalance by glycerol accumulation is a very deleterious event that leads to slow growth or even growth arrest.

At last, the GO biological process related to transmembrane transporters was underlined in the wild-type and calcineurin mutant strains; in the wild-type strain, more genes encoding transporters were repressed than induced, but in the *cnb*1Δ and *cna*1Δ mutants, 50 transporters were induced. The largest groups of DEGs are amino acids and sugar transporters (Figure 6C). Sugar transporters were mostly repressed or unaffected in the wild-type and *gpp*2Δ strains but induced or unaffected in the calcineurin mutants. Also, the ATP-binding cassette category (ABC transporters) was mostly repressed in the wild type and induced in the mutants. These results are consistent with our previous observations that permease genes are induced in the *gpp*2Δ mutant. The phenotypic data show that amino acids, such as proline, can rescue the sensitive phenotype of the *gpp*2Δ [21].

### 3.6. Oxidative Stress Response and Glutathione Metabolism Are Induced in cna1Δ, cnb1Δ, and gpp2Δ Mutant Strains

**Figure 7:**
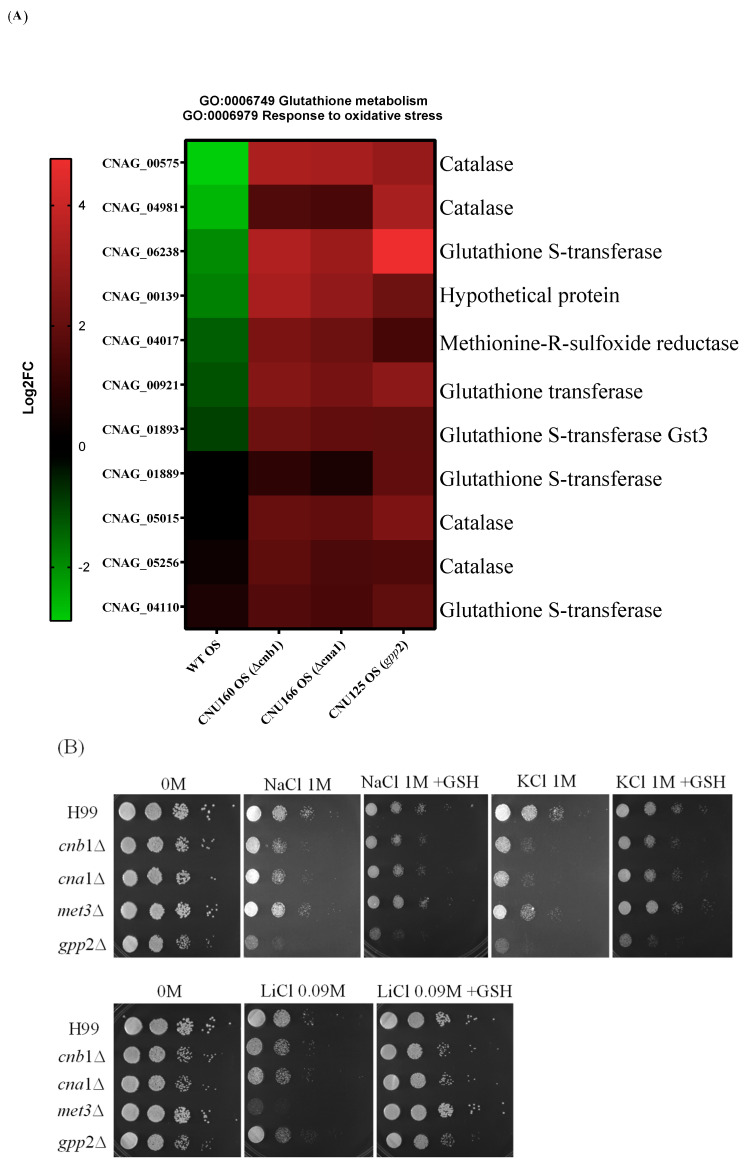
(**A**) A heat map of DEGs categorized under oxidative stress response (GO:0006979) and glutathione metabolism (GO:0006749); (**B**) the growth phenotypes of wild-type (H99) and *cnb1*Δ, *cna1*Δ, *met3*Δ, and *gpp2*Δ mutant strains cultured in YEPD medium containing NaCl, KCl (1 M each), or LiCl (0.09M) and supplemented with glutathione (10 mM).

Transcriptome analysis revealed that oxidative stress (GO:006979) and glutathione metabolism (GO:0006749) are induced in all three mutant strains (*cna*1Δ, *cnb*1Δ, and *gpp*2Δ) compared to the wild type (Figure 7A) during cationic stress. This observation implies that the inability to cope with cationic stress must cause reactive oxygen species to accumulate, requiring a response, which leads to the induction of 11 genes related to oxidative stress: 4 catalase genes and 5 genes related to glutathione metabolism (Figure 7A). In agreement with this result, we asked whether glutathione could also affect the growth rate in the presence of cationic stress. As shown in Figure 7B, 10 mM glutathione relieved the growth effects caused by NaCl, KCl, and LiCl. In addition, the best growth rate recovered with glutathione was observed for LiCl.

### 3.7. Calcineurin Affects the Expression of Fungal-Specific Zinc-Cluster Transcriptional Factors

**Figure 8:**
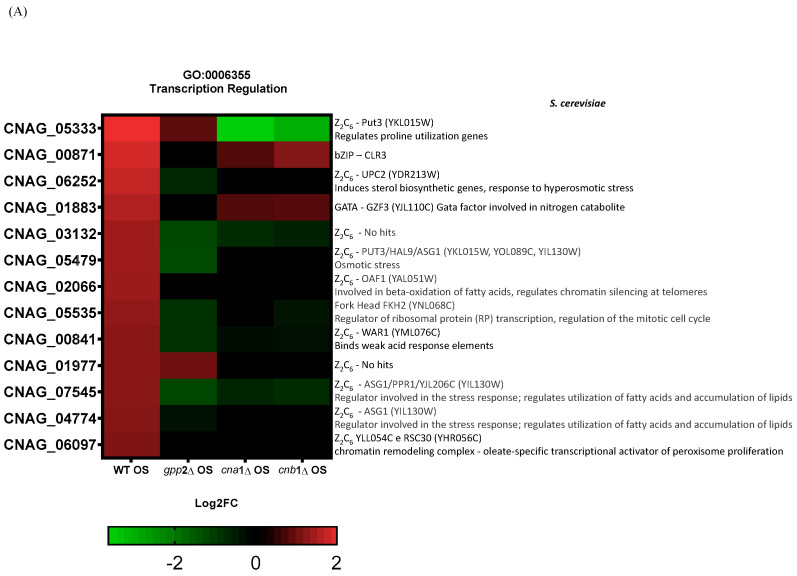

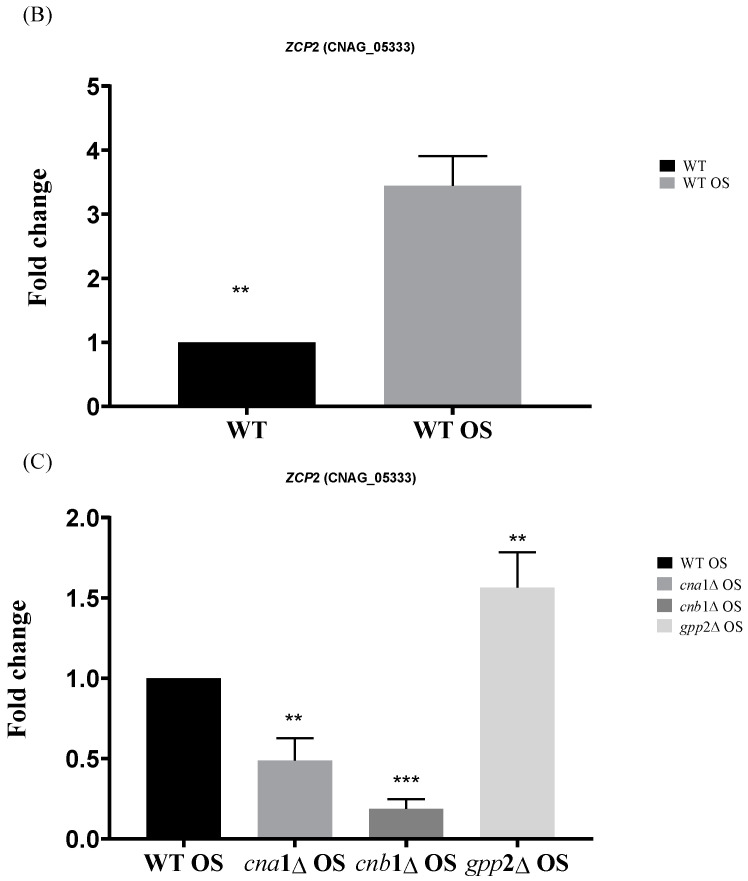
(**A**) A heat map of the DEGs categorized under transcription regulation (GO:0006355) in the wild type and mutants exposed to cationic stress. On the right side is the manual annotation based on the Saccharomyces genome database (SGD) and FungiDB. (**B**) The expression of CNAG_05333 (*ZCP*2 gene) by qPCR in the wild type (WT) and wild type under cationic stress (WT OS) and (**C**) CNAG_05333 (*ZCP*2) expression in *cna*1Δ, *cnb*1Δ, and *gpp*2Δ strains relative to the wild type during cationic stress by qPCR. Statistical differences tested by a one-way ANOVA test, ** *p* < 0.002 and *** *p* < 0.0002 (**C**).

Lev et al. (2012) reported that the *crz*1Δ mutant is not sensitive to cationic stress, but *cna*1Δ is, suggesting that other transcription factors may be post-translationally modified by calcineurin in response to cationic stress, or calcineurin may directly or indirectly induce one or more transcription factors at the transcriptional level in *C. neoformans* [14]. Therefore, it is reasonable to propose that, since calcineurin is a signaling complex, the disabling of its signaling pathway may have consequences for the induction of transcription factor genes. We directly searched for differential *CRZ*1 expression in the transcriptomes generated in this work, but *CRZ*1 expression levels were unaffected by the presence of NaCl in the wild type, and no differential expression was detected for *CRZ*1 in *cna*1Δ or *cnb*1Δ under this condition, suggesting that *CRZ*1 does not respond directly to cationic stress. However, a comparison of the wild-type and *gpp*2Δ strains highlighted the category (GO:0006355) related to the regulation of transcription, where 14 transcription factors were induced in the wild type and repressed in the *GPP*2 deletion mutant in the presence of NaCl. Of the 14 transcription factors, 11 belong to the Zinc-Cluster class of proteins, which are fungal-specific. The other three are BZip (CNAG_00871), GATA (CNAG_01883), and Fork-Head (CNAG_05535) transcription factors. The analysis was expanded to the *cna*1Δ and *cnb*1Δ strains, showing that all three mutants failed to induce most of the 14 putative transcription factors (heat map in Figure 8A). In the *gpp*2Δ strain, only two Zinc-Cluster proteins continued to be induced compared to the wild type during cationic stress, but to a lesser extent than in the wild type (CNAG_01977 and CNAG_05333). In the calcineurin mutants, only the GATA factor and one BZip continued to be induced. All other transcription factors were either not induced or repressed. In this case, the most repressed were CNAG_05333, CNAG_03132, CNAG_00841, and CNAG_07545. Out of 14 DEGs analyzed in the *gpp*2Δ strain, 7 transcription factors appeared repressed, all Zinc-Cluster transcription factors, except for the Fork-Head (Figure 8A). Finally, an important hallmark of the transcription factors identified in this study is that five of them are involved in the stress response and especially in fatty acid utilization and lipid accumulation in *S. cerevisiae* (CNAG_06252, CNAG_05479, CNAG_07545, CNAG_04774, and CNAG_06097). The most strongly induced gene under this condition (CNAG_05333) was validated by quantitative PCR (qPCR) under cationic stress in the wild-type strain, confirming the expression pattern observed in RNA-seq (Figure 8B) and the calcineurin-dependent expression in Figure 8C. These observations allowed us to suggest that (i) cationic stress induces a set of Zinc-Cluster transcription factors in a calcineurin-dependent manner, and (ii) several of these transcription factors seem to be related to lipid metabolism in other organisms.

## 4. Discussion

Calcineurin signaling is a key mechanism of adaptation in *C. neoformans*; it coordinates the thermal stress response through transcription factor dephosphorylation, spatial localization changes, and global transcriptional responses, leading to changes in cell wall dynamics, growth, virulence, and drug resistance, among other stress responses. The best-known calcineurin target is the Zinc-Finger protein Crz1, which triggers a calcineurin-dependent transcriptional response [6]. However, phenotypic analysis indicates that calcineurin acts in a Crz1-dependent and Crz1-independent manner in *C. neoformans*, suggesting that the complex has diverse means of signaling and different targets [15]. Indeed, in *C. neoformans,* calcineurin targets go beyond transcription factors. Our previous work also showed that Cna1 physically interacts with the Cys3 transcription factor, ATP sulfurylase, and Gpp2, a phosphatase involved in glycerol biosynthesis and osmoregulation [19,20,21]. In this paper, we show that calcineurin mutants are mildly sensitive to cationic stress compared to *gpp*2Δ, which is highly sensitive to hyperosmotic shock. This difference is likely because glycerol biosynthesis can be induced at the transcriptional level by Hog1, which is presumably still active in the calcineurin mutants, allowing at least a partial cationic stress response at the transcriptional level [34], whereas glycerol biosynthesis cannot be accomplished without Gpp2 activity. However, *cna*1Δ and *cnb*1Δ mutants undergo low glycerol accumulation during cationic stress to the same degree as *gpp*2Δ, suggesting that one reason why calcineurin mutants are sensitive to cationic stress is the inability to accumulate glycerol, as shown in Figure 1A,B, due to post-translational modification in the Gpp2 protein, which leads to its accumulation as a lower-molecular-weight protein compared to the wild type and the lack of proper subcellular localization (Figure 2 and Figure 3).

In *S. cerevisiae*, Hog1, calcineurin, and Torc2-Ypk1 signaling pathways share the role of promoting the cationic stress response [35,36,37,38]; however, to our knowledge, the direct link between calcineurin and glycerol biosynthesis has not been documented in *C. neoformans*, despite earlier reports on the osmosensitivity of calcineurin mutants in *C. neoformans*, which is also dependent on the serotype [10]. While having multiple signaling systems controlling a specific osmotic stress response is not without precedent, corroborating our findings as mentioned above, the mechanisms through which calcineurin takes part in the osmotic stress response are diverse in *C. neoformans* and *S. cerevisiae*, according to our data. In *S. cerevisiae*, part of the ion homeostasis response is calcineurin-regulated, since *ENA*1 transcription (Na^+^ and K^+^ influx pump), among others, is calcineurin-dependent [35,39]. On the contrary, in *C. neoformans, ENA*1 and *NHA*1 (Na^+^ efflux pump) expression have been reported as Hog1-dependent [34,40]. We searched our transcriptomes under cationic stress for these genes, and the data indicated that calcineurin does not regulate these genes in *C. neoformans* as it does in *S. cerevisiae*.

In spite of the lack of evidence on the transcriptional control of calcineurin over cationic stress, similar to what has been found in *S. cerevisiae*, calcineurin mutants in *C. neoformans* are sensitive to cationic stress (Figure 1). Moreover, physical and functional links between calcineurin and Gpp2 have been found [17,19]. In the present work, we showed two important modifications of the Gpp2 protein linked to calcineurin activity: lower-molecular-weight Gpp2 protein (Figure 2) and abnormal localization (Figure 3) were observed in calcineurin mutant background strains. Also, our data suggest that the interaction between calcineurin and Gpp2 is important to glycerol accumulation, which, once broken, leads to growth arrest in response to cationic stress. Our data do not allow us to conclude whether the modifications caused by calcineurin to the Gpp2 protein are direct or indirect or to deduce the nature of the modification. The fact that Gpp2 interacts physically with Cna1 suggests a direct interaction [19]. Similarly, the Tor pathway plays an important role in the cationic stress response in yeast [38]. One important post-translational mechanism is the regulation of Ypk1-Torc2-dependent kinase, which, in turn, leads to the dephosphorylation of Gpd1 during osmotic stress, rendering this protein active and, therefore, triggering glycerol accumulation [41]. Apparently, *S. cerevisiae* and *C. neoformans* use different signaling apparatuses to post-translationally regulate the glycerol biosynthetic pathway at different steps (Figure 5A) to guarantee efficient and quick regulation during cationic stress.

Calcineurin and Crz1 localize to stress granules or P-bodies during heat stress [18], a localization pattern very similar to the one observed here for Gfp-Gpp2 (Figure 3). While our data do not allow us to conclude that Gpp2 is a resident protein of P-bodies, we can speculate that once it physically interacts with Cna1 and is concentrated in cytoplasmic puncta during cationic stress, it may migrate to stress granules in a calcineurin-dependent fashion. However, this hypothesis remains to be further explored.

The compilation of previous and present data indicates that Gpp2 is a target of calcineurin, which is modified downstream of this signaling complex at the post-translational level, triggering glycerol biosynthesis [19,21,22]. In addition, *C. neoformans* calcineurin does not seem to activate the main transcriptional targets like in *S. cerevisiae*, as mentioned before, suggesting that it may cause a different transcriptional response that has not been described. Under these circumstances, we aimed to learn more about the global transcriptional response generated during cationic stress when cells are unable to undergo glycerol biosynthesis. The results highlight that cationic stress causes a general carbohydrate metabolism adaptation in wild-type and glycerol-deficient mutant strains, highlighting changes in cell wall maintenance and chitin metabolism in all strains, which is in line with required modifications on the cell wall surface that are important for maintaining cell wall integrity and turgor during stress [42,43]. However, carbohydrate metabolism is still very divergent between wild-type and glycerol-deficient strains (Figure 6A); the calcineurin and *gpp*2Δ mutants have a metabolic flow toward glyconeogenesis, glycosyl hydrolases, pentose phosphate pathway, and amino sugar and glycogen mobilization, highlighting the need for extra energy derived from alternative carbon sources during glycerol deficiency. This reinforces the link between cell wall integrity and carbon metabolism, which seem to be key factors when glycerol biosynthesis fails, probably by channeling the rescued alternative carbon to β-glucan biosynthesis. This model is supported by our observation that glycerol-deficient mutants have higher expression levels of glycogen phosphorylase (*S. cerevisiae GPH*1 gene, CNAG_06666) and UDP-glucose pyrophosphorylase (*S. cerevisiae UGP*1, CNAG_02748), which are key steps in β-glucan metabolism [44,45].

Furthermore, it is extremely important to raise the point that during cationic stress, the wild type’s metabolism flows toward glycerol biosynthesis, with *GPD* and *GPP* genes being induced (Figure 5); on the contrary, in the glycerol-deficient mutants, metabolism flows to glycerol-3-phosphate, which probably serves as an intermediate metabolite to both glycerolipid biosynthesis and glycolysis, with *GPT*2/*SCT*1 and *GUT*2 being highly induced (respectively), suggesting that glycerolipid biosynthesis is favored, probably to contribute to plasma membrane dynamics during stress. In yeast, the proper cell membrane composition affects membrane proteins, such as ATP-driven proton pumps, which, in turn, require specific lipid raft composition and abundance for correct efflux and influx activity, culminating in an adequate stress response [46].

Translation is highly repressed in glycerol-deficient mutants (Figure 6), which is consistent with the low growth in calcineurin mutants and growth arrest in *gpp*2Δ (Figure 1). In *C. neoformans*, other signaling systems that affect the osmotic stress response, such as Hog1 and Ras1, cause slow growth or growth arrest in different conditions, such as cationic, osmotic, oxidative, and thermal stresses, suggesting that this response probably aims to achieve a balance and save cellular energy to be expended on the very best transcriptional response [28,42,47,48,49].

Besides carbon metabolism, lipid and glycerol biosynthesis, and translation, membrane transport proteins are a major overexpressed gene category (Figure 6). This result is consistent with our earlier report in the literature, where many transmembrane genes were induced, and compatible solutes, such as amino acids, can compensate for glycerol deficiency [21,29,50,51,52].

It is interesting to point out that oxidative stress response and glutathione metabolism genes are induced under cationic stress when glycerol biosynthesis is blocked (*gpp*2Δ, *cna*1Δ, and *cnb*1Δ strains) compared to the wild type (Figure 7). Several oxidative stress genes are under the control of the Hog1 pathway [34]. Therefore, it is possible that calcineurin and Gpp2 do not directly control these genes. Still, the lack of glycerol and the inability to cope with cationic stress may lead to the accumulation of reactive oxygen species that actually activate the Hog1 pathway and the oxidative stress response (Figure 7). The best example here is the catalase gene (CNAG_00575, Figure 7), which is well known as the target of the Hog1 pathway [34], but it is repressed in the wild type under cationic stress and induced in all glycerol-deficient mutants in this study. Additionally, consistent with an important role in the oxidative stress response during cationic stress, glutathione supplementation in the medium partially rescued the slow-growth phenotype of the mutants. Other oxidative stress genes are under the control of Hog1, but they did not show differential expression in this work, suggesting that the depletion of glycerol triggers different oxidative stress response genes. Signaling systems, like Hog, Ras, and CWI (cell wall integrity response) also trigger the oxidative stress response, suggesting that ROS (reactive oxygen species) are deleterious enough to the cell that they deserve redundant signaling systems to counter act their effects [42,53].

Finally, transcriptome analysis revealed a very interesting group of Zinc-Cluster proteins. Most of them failed to be induced under glycerol depletion (Figure 8), and one of them (CNAG_05333) is calcineurin-dependent but nearly Gpp2-independent, meaning that calcineurin signaling controls a set of novel stress-responsive transcription factor genes. It is noteworthy that several of these genes have been identified in *S. cerevisiae* as transcription factors that regulate fatty acid utilization, lipid biosynthesis, peroxisome proliferation, and ultimately, the stress response since the deletion of several of them leads to multiple-stress sensitivity [54].

**Figure 9:**
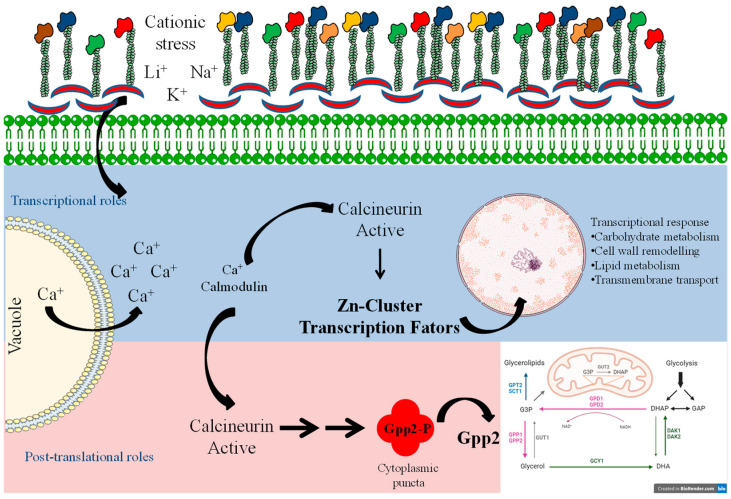
Model summarizing the signaling system of calcineurin and Gpp2 during cationic stress. The blue and pink shades depict the transcriptional and post-translational roles of calcineurin in cationic stress, respectively.

Taken together, these results provide evidence that calcineurin causes post-translational modifications in Gpp2 phosphatase that are necessary for its subcellular localization at cytoplasm puncta (Figure 9). The interaction between calcineurin and Gpp2 is necessary for glycerol accumulation and proper growth under cationic stress in *C. neoformans*. In addition, in this study, we have obtained transcriptional profiles of glycerol-deficient strains and identified calcineurin’s transcriptional roles in glycerol biosynthesis. The data compilation shown in Figure 9 allowed us to suggest a mechanism of cellular adaptation to low glycerol: in this case, the metabolic flow is toward alternative carbon sources’ utilization, such as glycerolipid and β-glucan biosynthesis, solute transport across the plasma membrane, growth arrest, and an oxidative stress response under cationic stress and glycerol depletion. Also, a group of putative Zinc-Cluster transcription factors have been associated with glycerol deprivation, suggesting they act on fatty acid metabolism and peroxisome proliferation to adapt yeast cells to cationic and oxidative stress. However, how these transcription factors act in *C. neoformans* is not known at this point. Still, this work has certainly opened an avenue for further investigation on a very important topic for pathogenesis and virulence, since the stress response is at the center of pathogen adaptation in the host.

## Figures and Tables

**Table 1:** Number of DEGs (up- and down-regulated genes) in wild type and mutant transciptomes (comparison).

Comparison	Up-Regulated DEGs	Down-Regulated DEGs
Wild type OS vs. Wild type	433	544
*cnb*1ΔOs vs. Wild type OS	815	376
*cna*1ΔOS vs. Wild type OS	760	315
*gpp*2Δ OS vs. Wild type OS	1396	1226

## Data Availability

All the relevant data in the paper are reported in Supplementary Information files. RNA-seq raw data is available at NCBI SRA database under accession number PRJNA1120105.

## Supplementary Materials

The following supporting information can be downloaded at: https://www.mdpi.com/article/10.3390/jof10080531/s1, Figures S1–S9: western blot original images; Figures S10–S19: microscopy original images; Table S1: plasmid list; Table S2: strain list; Table S3: primer list.
