# Supplementary material for: Transcriptional and Post-Translational Roles of Calcineurin in Cationic Stress and Glycerol Biosynthesis in Cryptococcus neoformans
Source: J Fungi (Basel). 2024 Jul 30;10(8):531. doi: 10.3390/jof10080531 (PMC11355346; doi:10.3390/jof10080531)
Supplement: Supplementary file 1 [file jof-10-00531-s001.zip › jof-3076507-supplementary.pdf]

Transcriptional and post translational roles of calcineurin in cationic stress and glycerol biosynthesis in *Cryptococcus neoformans*

Authors

Ronaldo Silva Santos [1], Gabriel Martins-Silva [1], Adrián Adolfo Álvarez Padilla [1], Mateus Possari [1], Sérgio Donnantuoni Degello [1], Otávio J. Bernardes Brustolini [2], Ana Tereza Ribeiro Vasconcelos [2], Marcelo Afonso Vallim [1] and Renata C. Pascon [1,*]

[1] Universidade Federal de São Paulo, Campus Diadema, Rua São Nicolau, 210, Diadema 09913-030, SP, Brazil

[2] Laboratório Nacional de Computação Científica—LNCC, Labinfo-Laboratório de Bioinformática, Petrópolis 25651-075, RJ, Brazil

* Corresponding author: renata.pascon@unifesp.br

Keywords: *Cryptococcus neoformans*; calcineurin; glycerol phosphate phosphatase; osmotic stress; cationic stress, *GPP*2

Supplementary Figure S1: Original membrane image presented in the western blot, after 2 hours of induction at NaCL (Figure 2A). The first 3 lanes were cropped.

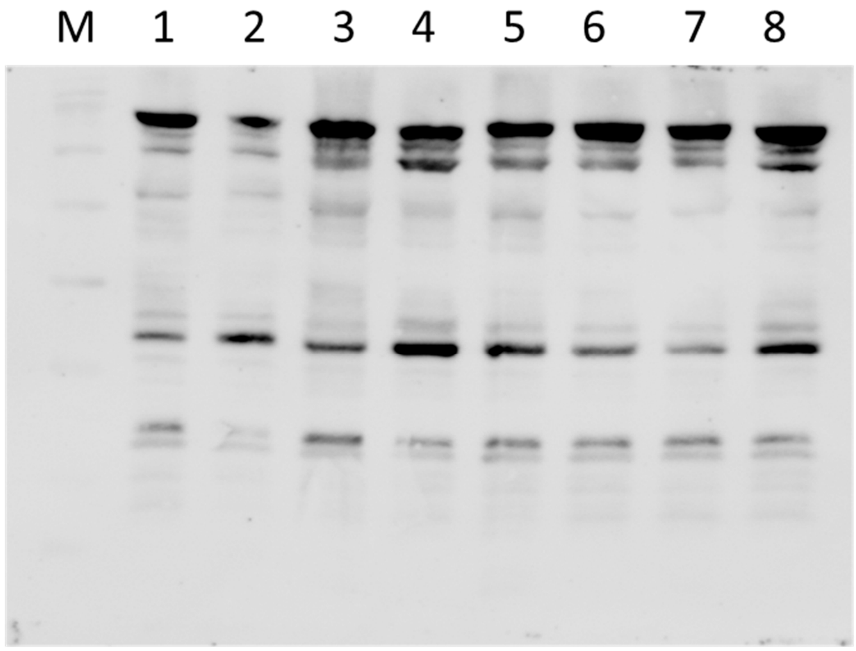

M- Molecular weight marker

1- CNU151 0M

2- CNU151 1.5M

3- CNU151 0M BR1

4- CNU151 1.5M BR1

5- CNU151 0M BR2

6- CNU151 1.5M BR2

7- CNU151 0M BR3

8- CNU151 1.5M BR3

*BR- Biological Replica

Supplementary Figure S2: Original western blot membrane that originated Figure 2A, detecting Gfp-Gpp2. Anti-Gfp antibody.

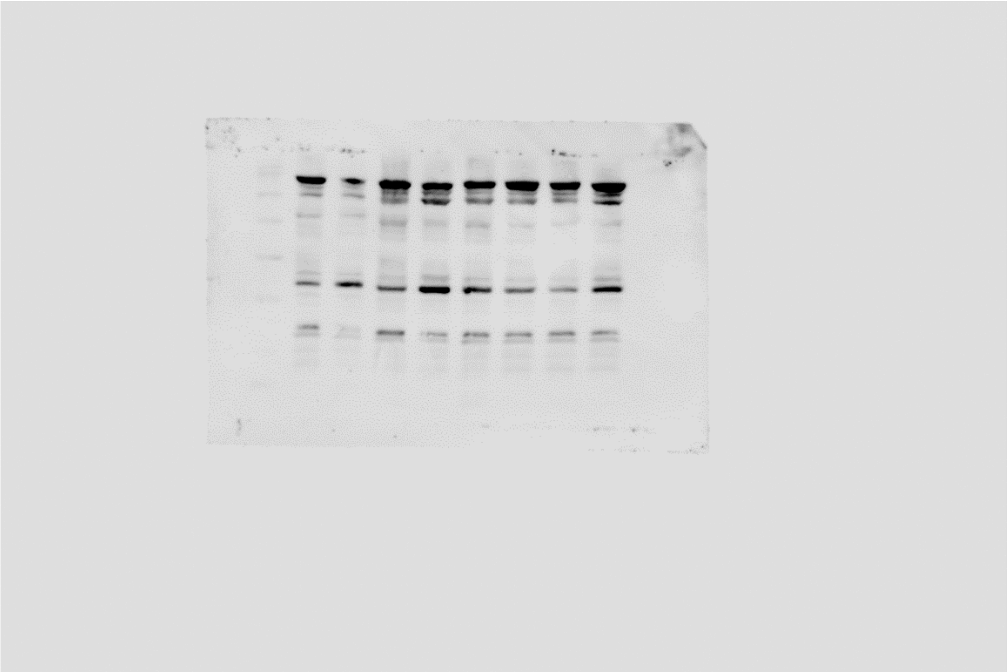

Supplementary Figure S3: Original western blot membrane that originated Figure 2A - normalizer Histone H3. Anti-Histone antibody.

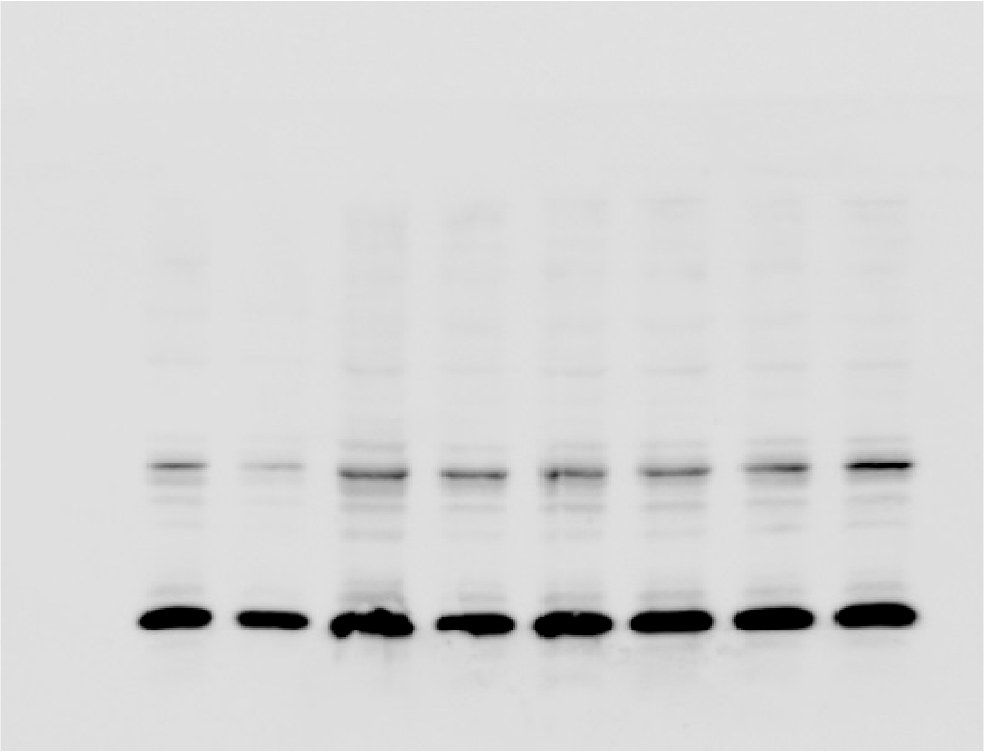

Supplementary Figure S4: Original membrane image presented in the western blot, after 2 hours of induction at NaCl (Figure 2B). The first 3 lanes were cropped.

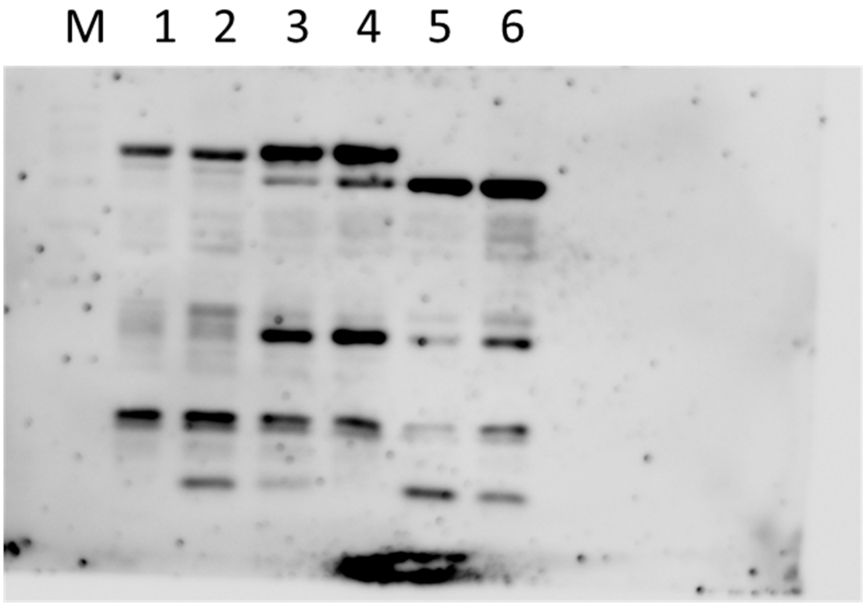

M- Molecular weight marker
1- CNU151 0M
2- CNU151 1.5M
3- CNU 189 0M
4- CNU189 1.5M
5- CNU193 0M
6- CNU193 1.5M

Supplementary Figure S5: Original western blot membrane that originated Figure 2B, detecting Gfp-Gpp2. Anti-Gfp antibody.

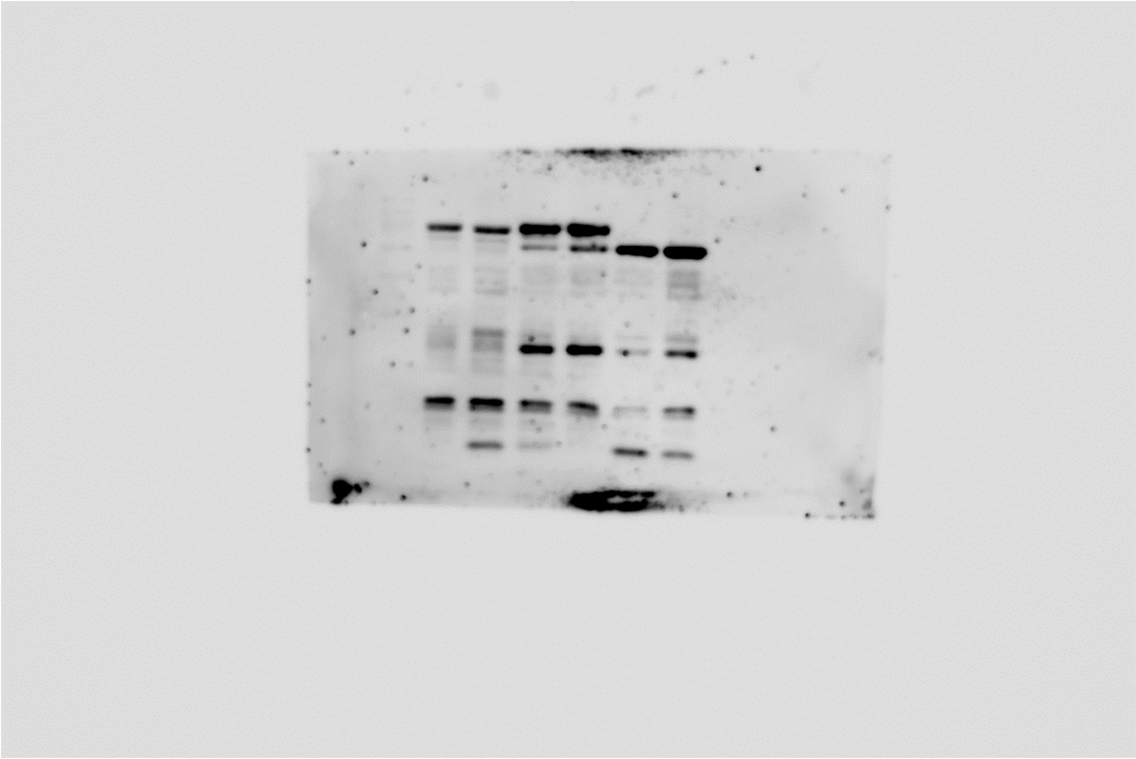

Supplementary Figure S6: Original western blot membrane that originated Figure 2B - normalizer Histone H3. Anti-Histone antibody.

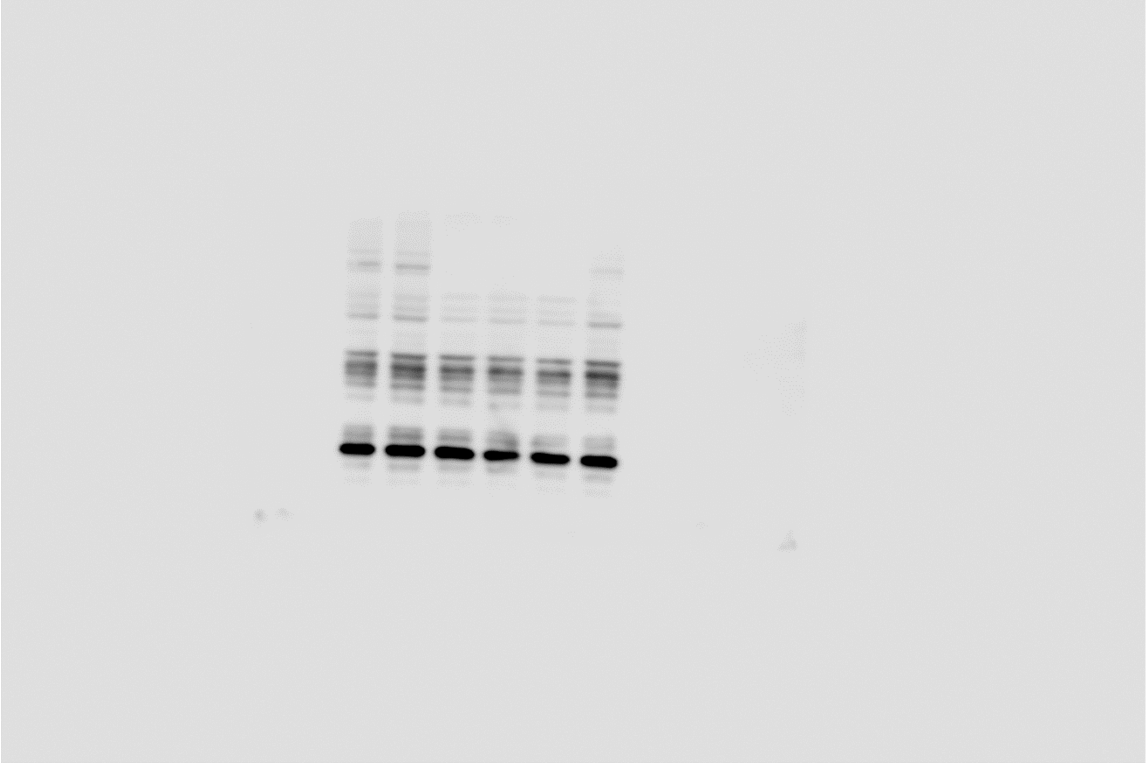

Supplementary Figure S7: Original membrane image presented in the western blot, after 0, 2 and 4 hours of exposure on cyclosporine A CsA (Figure 2C).

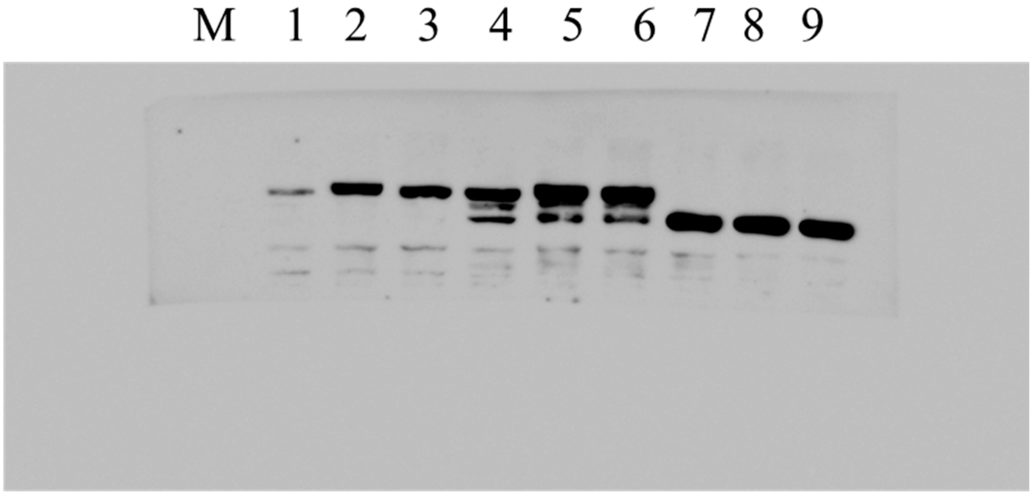

M- Molecular weight marker

1- CNU151 0h CsA

2- CNU151 2h CsA

3- CNU 151 4h CsA

4- CNU189 0h CsA

5- CNU189 2h CsA

6- CNU189 4h CsA

7- CNU193 0h CsA

8- CNU193 2h CsA

9- CNU193 4h CsA

Supplementary Figure S8: Original western blot membrane that originated Figure 2C, detecting Gfp-Gpp2. Anti-Gfp antibody.

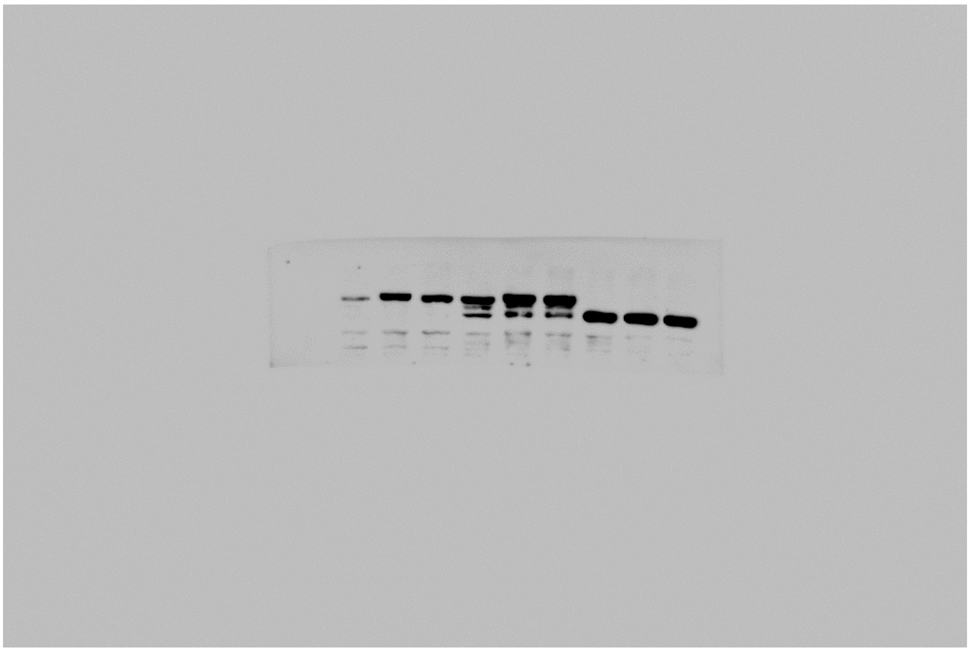

Supplementary Figure S9: Original western blot membrane that originated Figure 2C - normalizer Histone H3. Anti-Histone antibody.

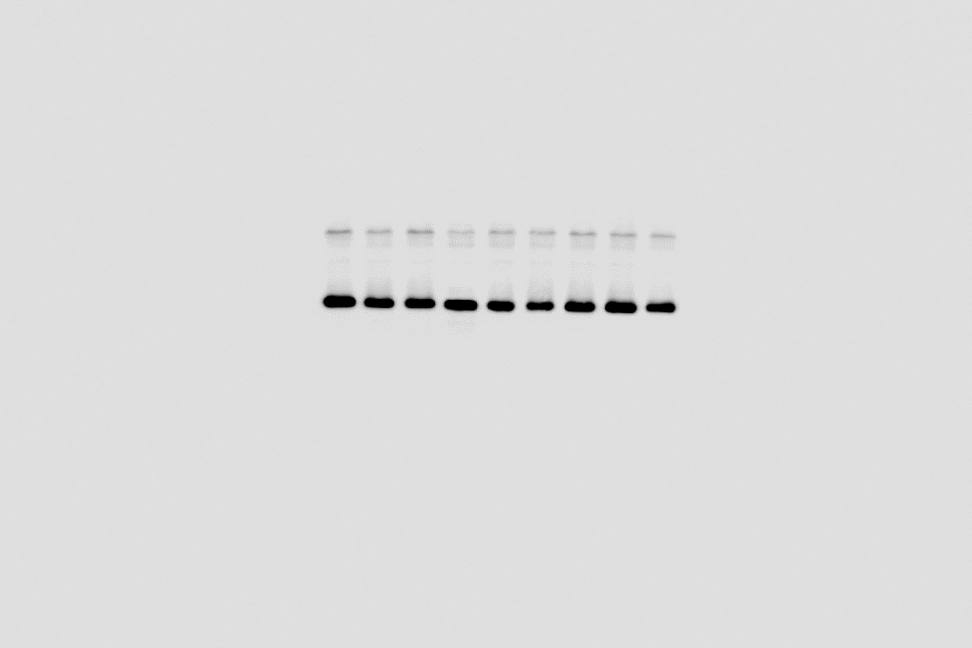

Supplementary Figure S10: Original microscopy image of Gfp-Gpp2 protein of the strain CNU151 (wild type) presented in the (figure 3A) after induction in 0M NaCl for 2 hours.

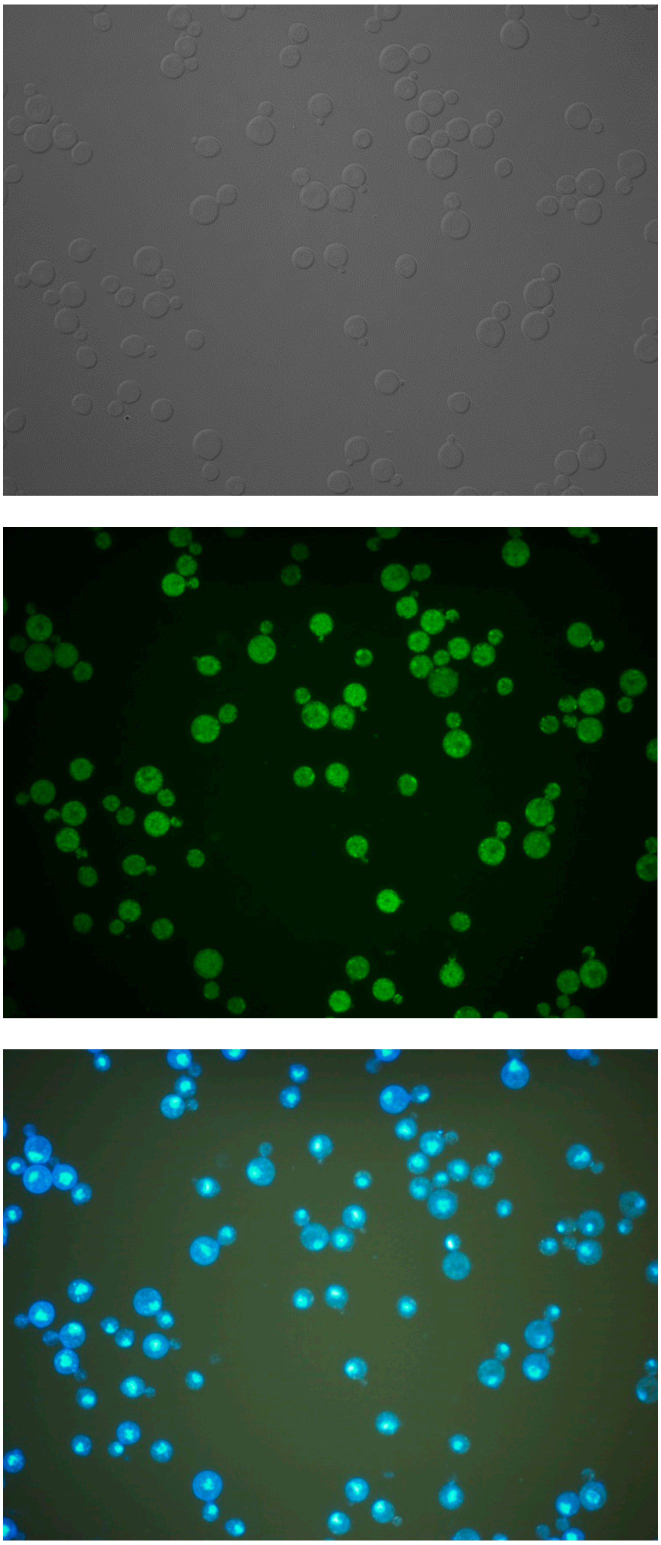

Supplementary Figure S11: Original microscopy image of Gfp-Gpp2 protein of the strain CNU151 (wild type) presented in the (figure 3A) after induction in 1,5M NaCl for 2 hours.

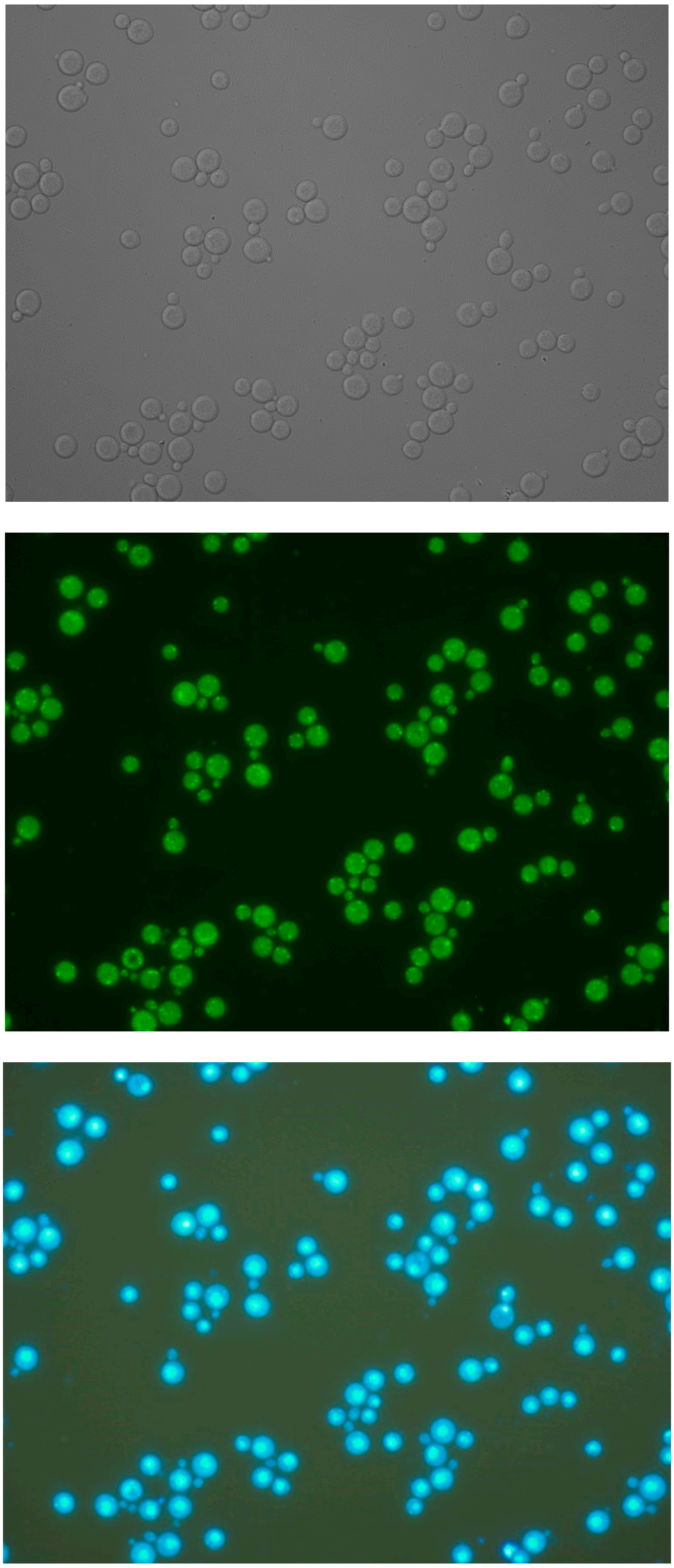

Supplementary Figure S12: Original microscopy image of Gfp-Gpp2 protein of the mutant strain *cnb*1Δ presented in the (figure 3C) after induction in 0M NaCl for 2 hours.

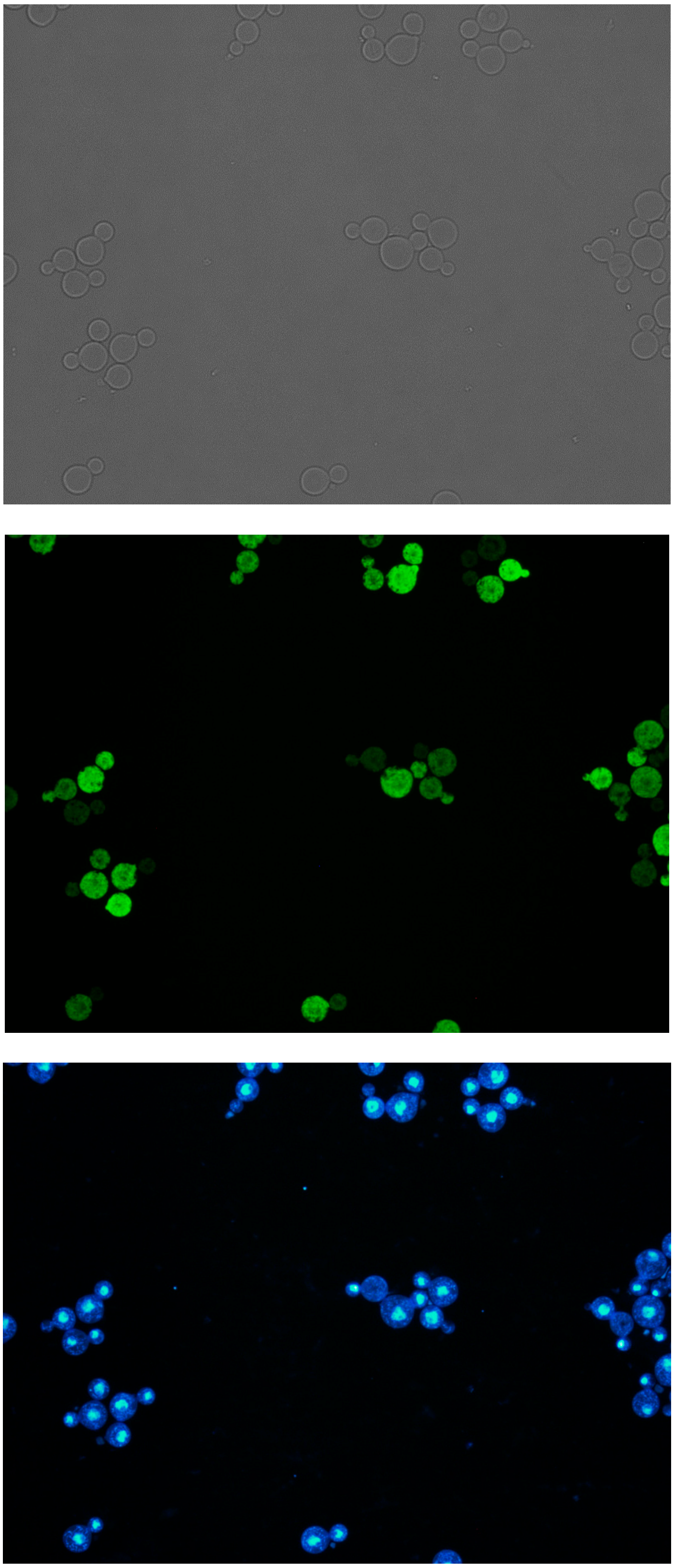

Supplementary Figure S13: Original microscopy image of Gfp-Gpp2 protein of the mutant strain *cnb*1Δ presented in the (figure 3C) after induction in 1,5M NaCl for 2 hours.

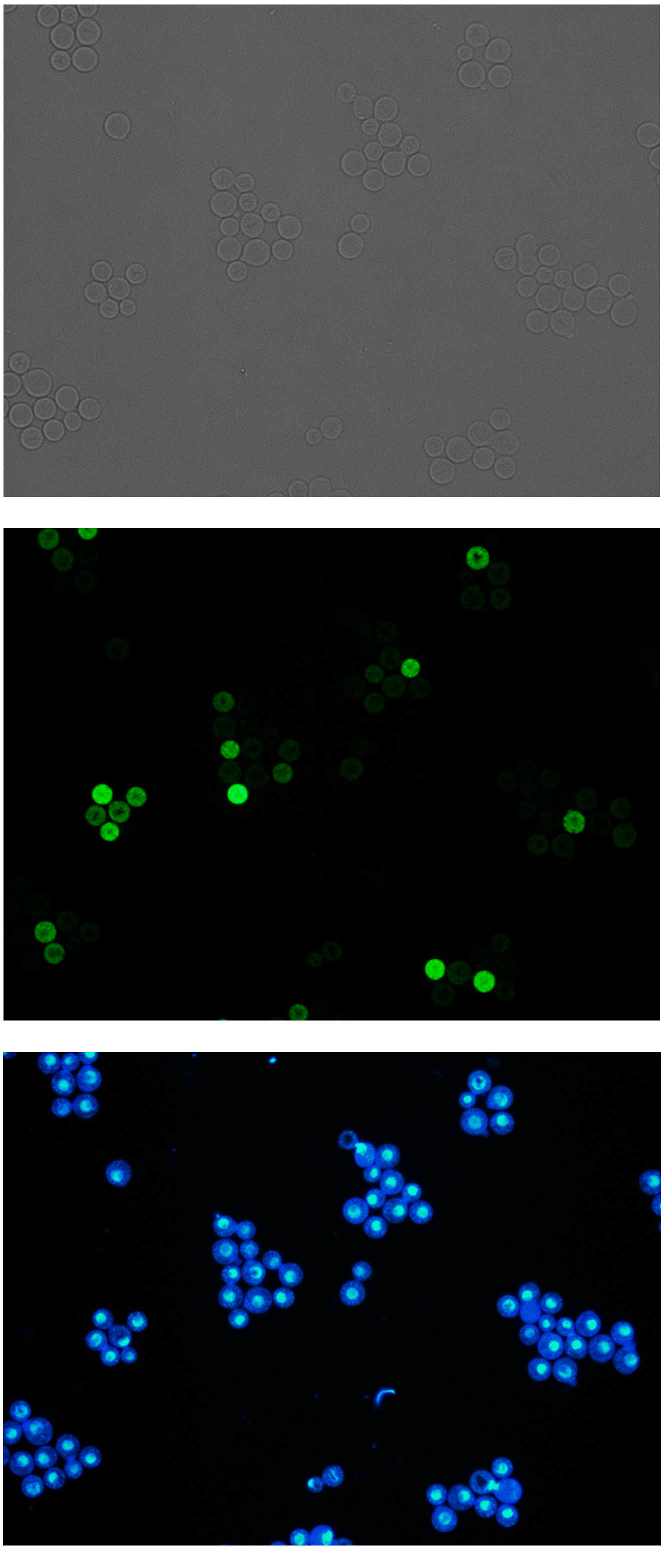

Supplementary Figure S14: Original microscopy image of Gfp-Gpp2 protein of the mutant strain *cna1Δ* presented in the (figure 3C) after induction in 0M NaCl for 2 hours.

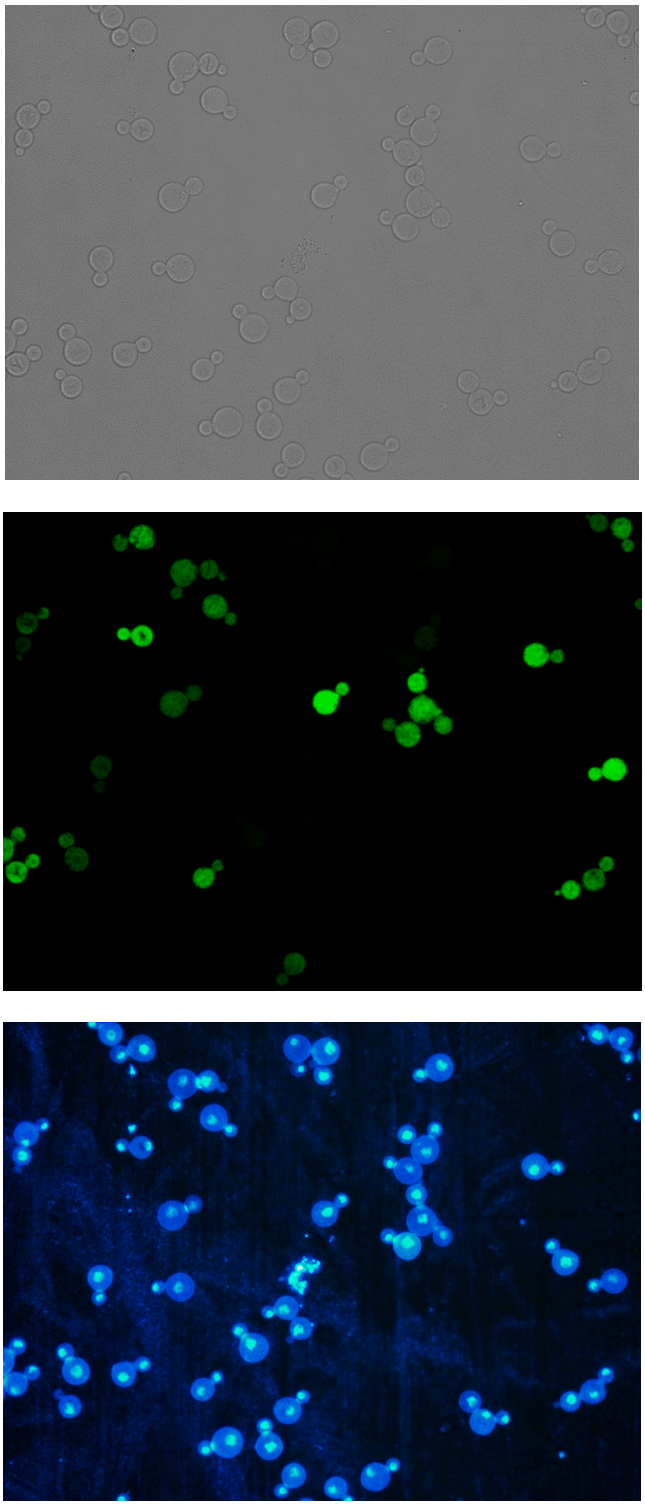

Supplementary Figure S15: Original microscopy image of Gfp-Gpp2 protein of the mutant strain *cna1*Δ presented in the (figure 3C) after induction in 1,5M NaCl for 2 hours.

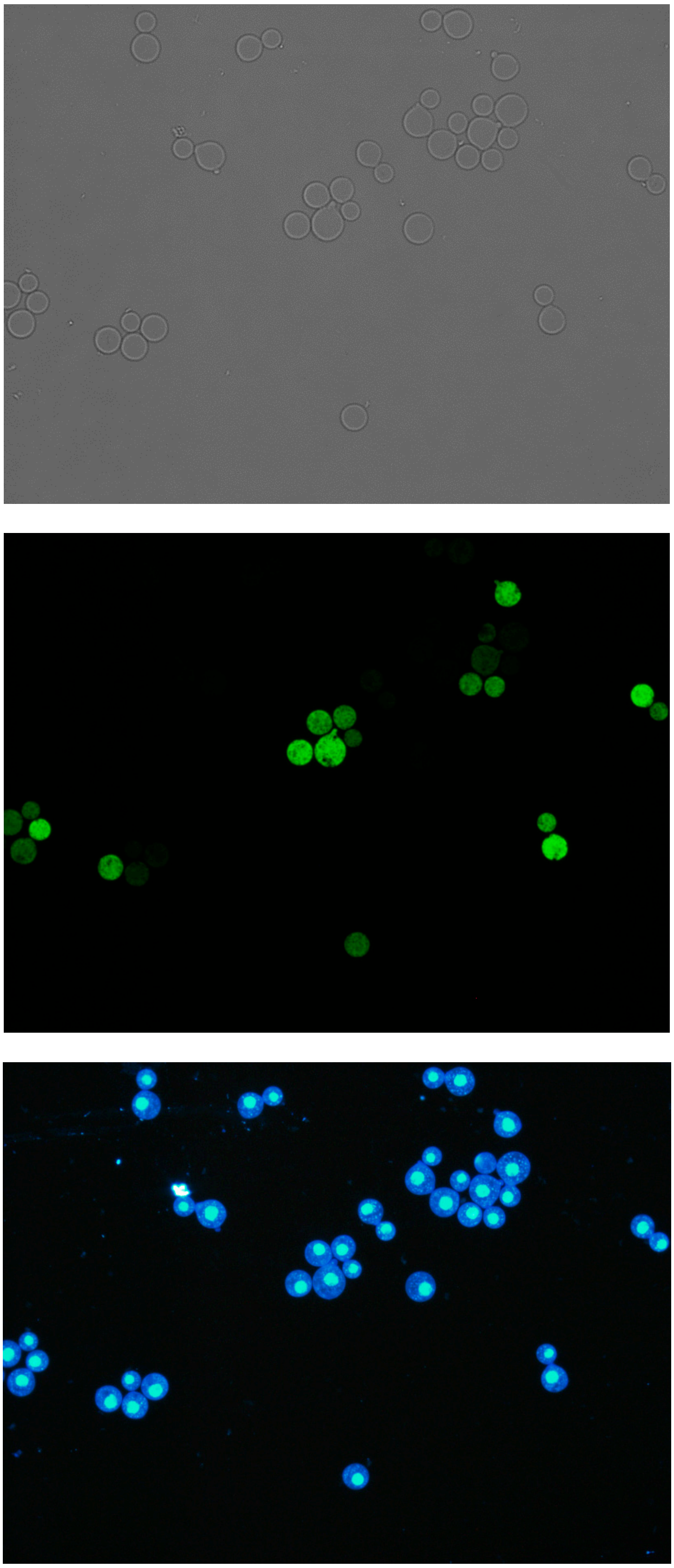

Supplementary Figure S16: Original microscopy image of Gfp-Gpp2 protein of the strain CNU151 (wild type) presented in the (figure 3D) after exposure with 0 µg/mL CsA for 2 hours.

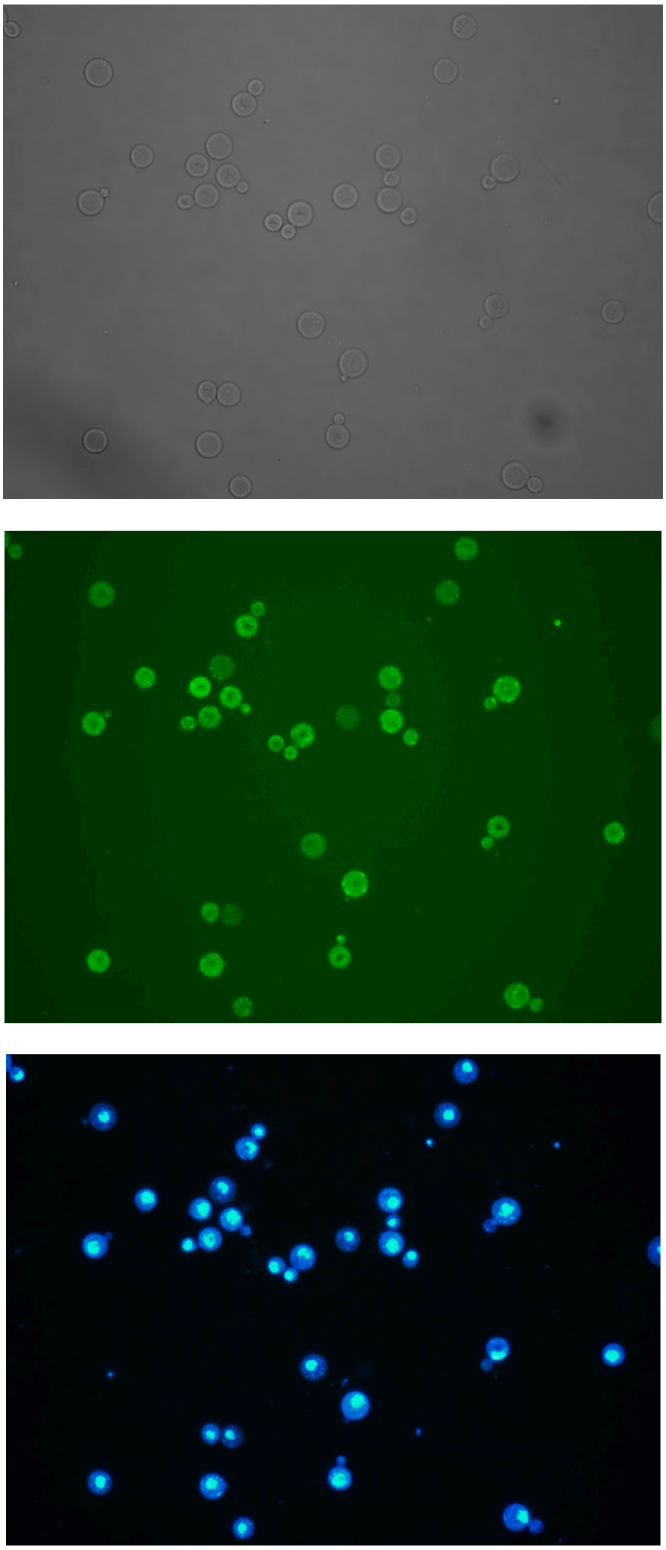

Supplementary Figure S17: Original microscopy image of Gfp-Gpp2 protein of the strain CNU151 (wild type) presented in the (figure 3D) after exposure with 100 µg/mL CsA for 2 hours.

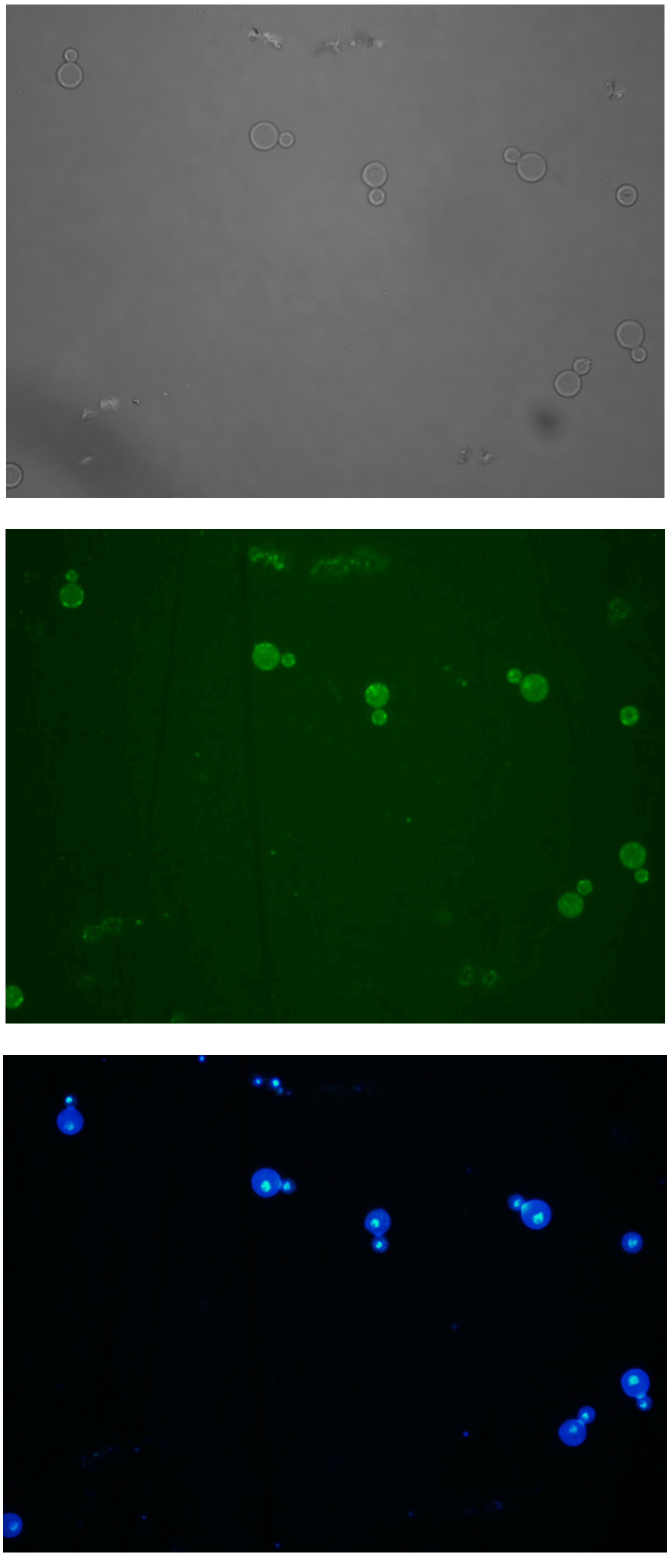

Supplementary Figure S18: Original microscopy image of Gfp-Gpp2 protein of the strain CNU151 (wild type) presented in the (figure 3D) after exposure with 0 µg/mL CsA for 4 hours.

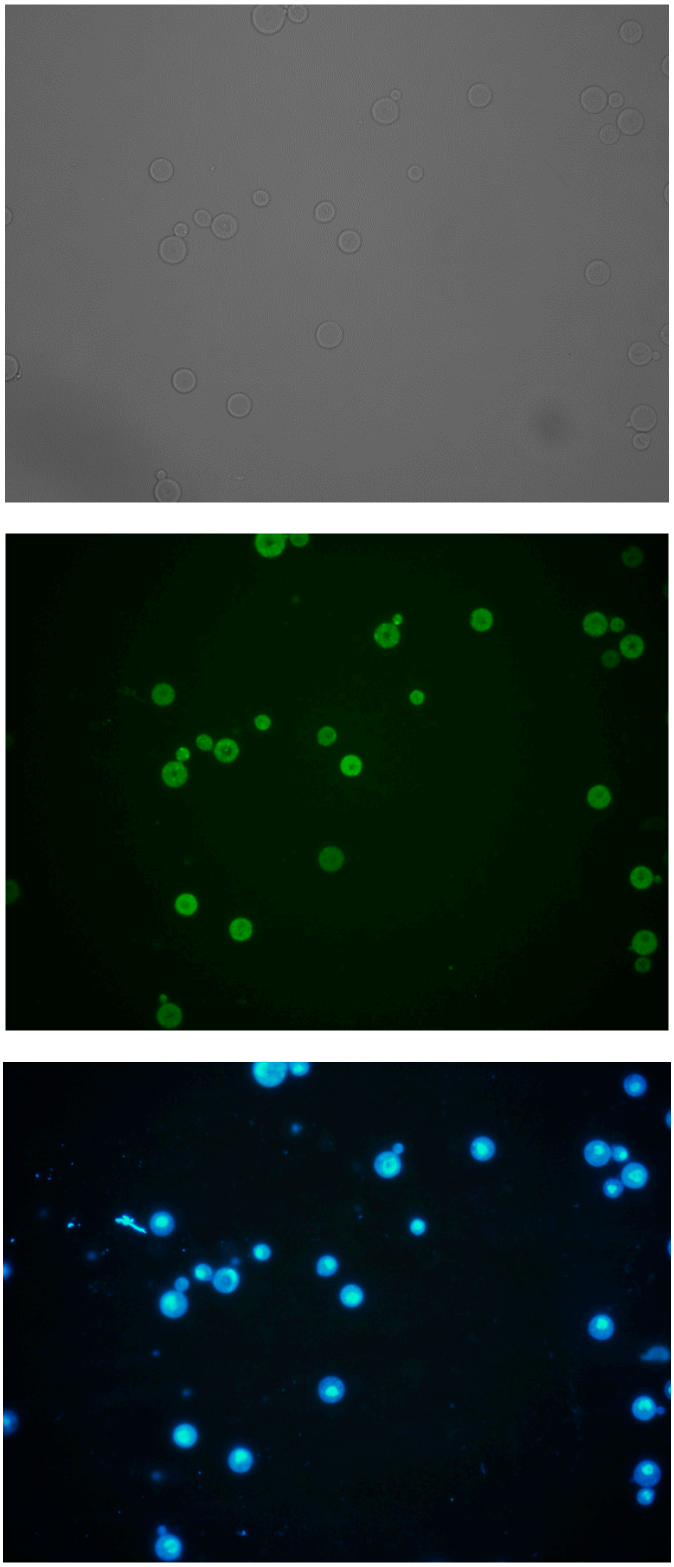

Supplementary Figure S19: Original microscopy image of Gfp-Gpp2 protein of the strain CNU151 (wild type) presented in the (figure 3D) after exposure with 100 µg/mL CsA for 4 hours.

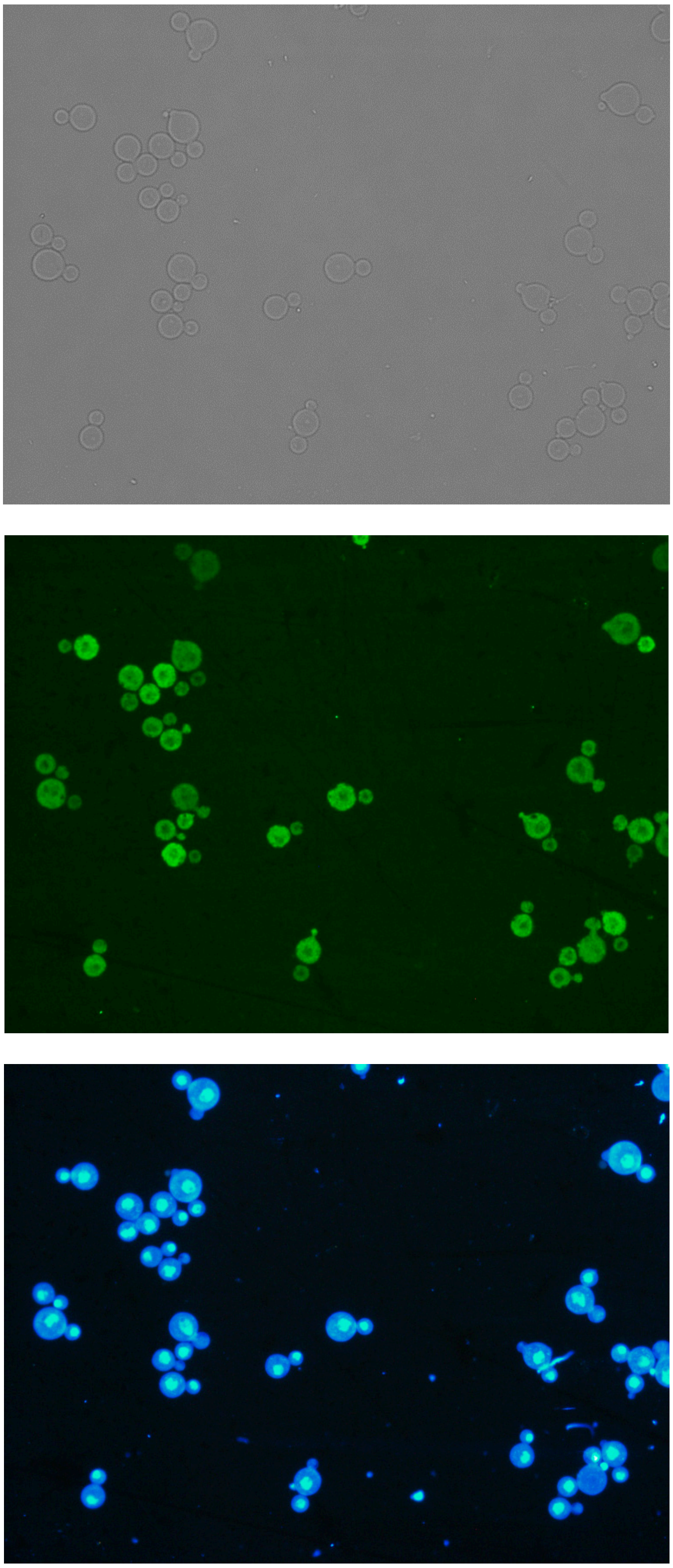

Supplementary Table S1: Plasmids used in this work.

Plasmid code	Vector	Gene	E. coli antibiotic resistance	Fungal Gene marker	Source
pRCP115	pCN19	*GFP:GPP2*	Amp/Kan	NAT	This work

Supplementary Table S2: Strains used in this work.

Strain Code	Background strain	Gene marker	Origen
		C. neoformans	
CNU151	H99	*GFP*:*GPP2*:Nat^R	This work
CNU080	H99	*GFP*::*CSY3* Neo^R	De Melo et al., 2019.
CNU160	H99	*cnb1Δ*:: Neo^R	This work
CNU166	H99	*cna1Δ*::Hph^R	Da Silva et al., 2023
CNU189	CNU160	*GFP:GPP2*::*cnb1*Δ	This work
CNU193	CNU166	*GFP:GPP2*::*cna1*Δ	This work
CNU153	CNU080	*met3Δ::*NAT^R	Da Silva et al.,2023
CNU 125	CNU080	*gpp2Δ::* Nat^R	Martho et al., 2019

Supplementary Table S3: Primers used in this work.

Primer	Sequence	F or R	Use
PRCP407	GTTCATGTACAGCTACACTTTGC	F	CNB1 deletion
PRCP408	CTCCAGCTCACATCCTCGCATGCAATAAGGCGGTATTGATGATG	R	CNB1 deletion
PRCP409	CATCATCAATACCGCCTTATTGCATGCGAGGATGTGAGCTGGAG	F	CNB1 deletion
PRCP410	GTATATGGGGTAGGAATGAGAAAGAAGAGATGTAGAAACTAGCTTCC	R	CNB1 deletion
PRCP411	GGAAGCTAGTTTCTACATCTCTTCTTTCTCATTCCTACCCCATATAC	F	CNB1 deletion
PRCP412	CGCAGGAAGTCGTTGGGAATC	R	CNB1 confirmation
PRCP472	GTACGAGCTCGGATCCATGTCCGTCTTCACCAAGTCTGCC	F	GFP-GPP2
PRCP473	GGCGGCCGTTACTAGTTGGCAGTCTTTATTCTTACCCAGC	R	GFP-GPP2
